# Radiosensitization of Breast Cancer Cells with a 2-Methoxyestradiol Analogue Affects DNA Damage and Repair Signaling In Vitro

**DOI:** 10.3390/ijms24043592

**Published:** 2023-02-10

**Authors:** Elsie Magdalena Nolte, Anna Margaretha Joubert, Laurence Lafanechère, Anne Elisabeth Mercier

**Affiliations:** 1 Department of Physiology, University of Pretoria, Pretoria 0007, South Africa; 2 Team Cytoskeleton Dynamics and Nuclear Functions, Institute for Advanced Biosciences, INSERM U1209, CNRS UMR5309, Université Grenoble Alpes, 38700 Grenoble, France

**Keywords:** cancer, radiation, 2-methoxyestradiol analogue, ESE-16, apoptosis, radiosensitization, DNA damage and repair, radiation-induced bystander effect, microtubule dynamics, actin

## Abstract

Radiation resistance and radiation-related side effects warrant research into alternative strategies in the application of this modality to cancer treatment. Designed in silico to improve the pharmacokinetics and anti-cancer properties of 2-methoxyestradiol, 2-ethyl-3-*O*-sulfamoyl-estra-1,3,5(10)16-tetraene (ESE-16) disrupts microtubule dynamics and induces apoptosis. Here, we investigated whether pre-exposure of breast cancer cells to low-dose ESE-16 would affect radiation-induced deoxyribonucleic acid (DNA) damage and the consequent repair pathways. MCF-7, MDA-MB-231, and BT-20 cells were exposed to sub-lethal doses of ESE-16 for 24 h before 8 Gy radiation. Flow cytometric quantification of Annexin V, clonogenic studies, micronuclei quantification, assessment of histone H2AX phosphorylation and Ku70 expression were performed to assess cell viability, DNA damage, and repair pathways, in both directly irradiated cells and cells treated with conditioned medium. A small increase in apoptosis was observed as an early consequence, with significant repercussions on long-term cell survival. Overall, a greater degree of DNA damage was detected. Moreover, initiation of the DNA-damage repair response was delayed, with a subsequent sustained elevation. Radiation-induced bystander effects induced similar pathways and were initiated via intercellular signaling. These results justify further investigation of ESE-16 as a radiation-sensitizing agent since pre-exposure appears to augment the response of tumor cells to radiation.

## 1. Introduction

Locoregional radiotherapy is an important component in the treatment regimens of various primary as well as secondary cancers. However, therapeutic radiation doses are limited by adverse reactions since non-cancerous cells are also irradiated [1]. Additionally, the development of radiation resistance due to fractionated regimens further reduces the therapeutic efficacy of the modality [2,3]. Two important strategies to overcome the above constraints include protecting the surrounding tissue from radiation damage and sensitizing tumor cells to radiation utilizing suitable anti-cancer drugs [4].

Radiation-induced deoxyribonucleic acid (DNA) double-strand breaks can be repaired via non-homologous end joining (NHEJ) or homologous recombination (HR) [5,6]. The majority of double-strand DNA breaks are repaired via rapid NHEJ, as HR is a much slower process. Damage to the cellular genome is detected by various DNA damage proteins, such as ataxia–telangiectasia-mutated (ATM), ataxia telangiectasia and Rad3-related (ATR), and DNA-dependent protein kinase (DNA-PK) [7]. Nuclear foci of H2AX are located along megabase chromatin domains which neighbor the sites of damage. H2AX is phosphorylated (γH2AX) by the aforementioned proteins and accumulates at the site of damage, serving as a docking site for DNA repair proteins involved in NHEJ [8].

The radiation-induced bystander effect refers to the biological consequences of radiation within non-radiated cells located in relative proximity to the directly exposed cells [9]. Due to signals received from radiated cells, non-irradiated cells may exhibit reactive biological responses such as alterations in gene expression and protein translation, changes in cell proliferation, and induction of apoptotic cell death [9]. This intercellular signaling is transmitted through soluble factors released by radiated cells and includes, amongst others, reactive oxygen species (ROS), reactive nitrogen species, cytokines, and micro-ribonucleic acid (microRNA) [10,11]. Alternatively, communication occurs through gap junctions between adjacent cells [12].

Hydroxylation of either the A ring into 2- or 4-hydroxy derivatives or the D ring at position 16α constitute the two main pathways of estrogen metabolism, mediated by various cytochrome P450 enzymes [13,14]. 2-Hydroxyestradiol is mostly converted to 2-methoxyestradiol (2-ME), whilst 4-hydroxyestradiol is further oxidized into genotoxic estrone(estradiol)-3,4-quinones [15]. 2-ME has demonstrated anti-cancer and anti-angiogenic properties by, amongst other mechanisms, binding to the colchicine site and disrupting the intracellular function and structure of microtubules [16,17]. However, low oral bioavailability and a poor pharmacokinetic profile due to glucuronidation curtailed the clinical application of 2-ME (clinical trials registered as Panzem^®^) [16,18,19,20]. To redress the compound’s shortfalls while simultaneously enhancing its cytotoxicity, our laboratory in silico-designed a sulfamoylated 2-ME analogue, namely 2-ethyl-3-*O*-sulfamoyl-estra-1,3,5(10)16-tetraene (ESE-16) [21]. Dehydration of the metabolically active C17 position of the D-ring aimed to decrease 17β-hydroxysteroid dehydrogenase-mediated metabolism. Addition of a sulfamate group at C3 of the A-ring promotes binding to carbonic anhydrase II within erythrocytes, thus bypassing the first-pass hepatic metabolism. These modifications resulted in a demonstrable increased oral bioavailability in murine models [22]. Additionally, preferential binding to carbonic anhydrase IX, which is overexpressed in the tumor microenvironment, would theoretically ensure more specific localization of ESE-16 to the tumor [21].

ESE-16 has proven cytotoxic effects at nanomolar concentrations in numerous cancer cell lines in vitro. The compound retained its cytotoxicity in a multidrug-resistant, P-glycoprotein efflux pump-overexpressing cell line [23] and induced both type I and type II programmed cell death [24,25]. ESE-16 initiates a dysregulated autophagic response, which may be attributed to dysfunctional trafficking of the autophagosomes due to abrogated microtubule dynamics [26,27]. As a microtubule-targeting agent causing a prolonged metaphase block, ESE-16 could potentially be used to expose cellular DNA to the damaging effects of radiation during this vulnerable stage of the cell cycle. [28,29].

The potential radiosensitizing effect of ESE-16 on tumor cells and the signaling pathways involved have not been fully investigated. As malignant cells are more susceptible to ESE-16 than normal cells (with and without radiation), this treatment offers a therapeutic preference that could be exploited clinically [30,31,32]. Hence, the aim of this in vitro study was to investigate whether a 24 h exposure of three breast cancer cell lines to a sub-lethal dose of ESE-16 prior to radiation would decrease long-term cellular survival, augment DNA damage, and/or impair the repair thereof. In addition, the possible radiation-induced bystander effect was investigated on untreated cells. Estrogen/progesterone receptor-positive (ER/PR+) human epidermal growth factor receptor 2 negative (HER2/Neu-) MCF-7, metastatic MDA-MB-231 (ER/PR-; HER2/Neu-), and BT-20 (ER/PR-; HER2/Neu-) tumorigenic human epithelial breast cancer cell lines were used for this study to eliminate any potential hormone receptor involvement. A pharmaceutical agent that induces cell death irrespective of receptor status, selectively spares non-neoplastic cells, and reduces radiation dose requirements could be advantageous in the treatment of these cancers.

## 2. Results

### 2.1. ESE-16 Exhibits Cytotoxicity at Nanomolar Concentrations

**Figure 1:**
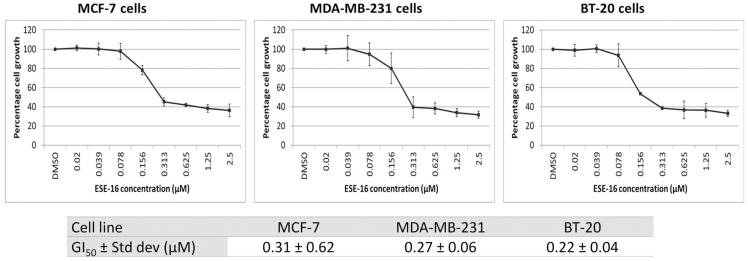
Dose-response curve to determine ESE-16 cytotoxicity in breast cancer cell lines. MCF-7, MDA-MB-231 and BT-20 cells were exposed to ESE-16 for 48 h. Cell viability is expressed as the percentage of cell growth calculated relative to the DMSO vehicle control. Standard deviations (STD) are represented by T-bars.

Dose-response curves revealed GI_50_ values of 0.31 ± 0.62 µM in MCF-7 cells, 0.27 ± 0.06 µM in MDA-MB-231 cells, and 0.22 ± 0.04 μM in BT-20 cells (Figure 1). These results indicated that cytotoxicity of ESE-16 was independant of cell receptor status.

### 2.2. ESE-16 Depolymerizes Microtubules and Affects Actin Fibers in Breast Cancer Cells

**Figure 2:**
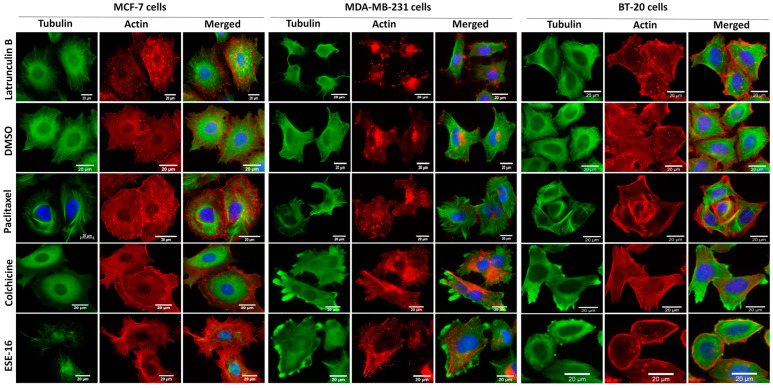
ESE-16 depolymerized cellular microtubules and disrupted actin morphology. An intact microtubule network (green) was observed in cells exposed to DMSO. Paclitaxel exposure resulted in stabilization of the microtubules, whereas colchicine exposure resulted in their depolymerization. MCF-7, MDA-MB-231, and BT-20 cells exposed to ESE-16 displayed tubulin staining similar to that of the colchicine-treated samples, with no detectable residual microtubules. Latrunculin B exposure provided a positive control for actin (stained red) depolymerization. Actin filaments displayed a radial arrangement in BT-20 cells after ESE-16 treatment, and thickened stress fibers in the other two cell lines. Cell nuclei were counterstained with DAPI (blue). Scale bars = 20 µm.

The effects of ESE-16 (at GI_50_ concentrations) on the cytoskeleton of the three breast cancer cell lines (24 h) were visualized with fluorescent microscopy employing an anti-tubulin antibody, phalloidin (actin staining), and 4′,6-diamidino-2-phenylindole (DAPI) (nuclear stain) (Figure 2). Cells exposed to DMSO served as the vehicle control and displayed intact microtubule networks (green) with most cells present in interphase. Actin staining (red) revealed normal stress fiber formation along the leading edges of the cells, polarizing the cells for movement. Cells exposed to paclitaxel served as a positive control for microtubule stabilization, while colchicine provided a positive control for microtubule depolymerization. Latrunculin B served as a positive control for actin depolymerization. MCF-7, MDA-MB-231, and BT-20 cells exposed to ESE-16 displayed similar changes as those treated with colchicine, adopting a rounded cell morphology with depolymerized microtubules. BT-20 cells displayed peripheral radial arrangements of actin filaments, with no evident polarity. The actin skeleton of ESE-16-exposed MCF-7 and MDA-MB-231 cells displayed an increase in stress fiber formation and disruption of cell polarity.

Thereafter, we established the lowest dose of ESE-16 that significantly affected cell viability (Supplementary data: section S2, Figures S1–S4). This dose was then used in assays to determine the possible radiosensitizing effect of ESE-16. In all experiments, no statistically significant differences were observed between the negative and vehicle control samples, indicating that DMSO had no detectable effect on cell viability under these experimental conditions.

### 2.3. Treatment with ESE-16 Prior to Radiation Induces a Metaphase Block and Increases Early Apoptotic Cell Death

**Figure 3:**
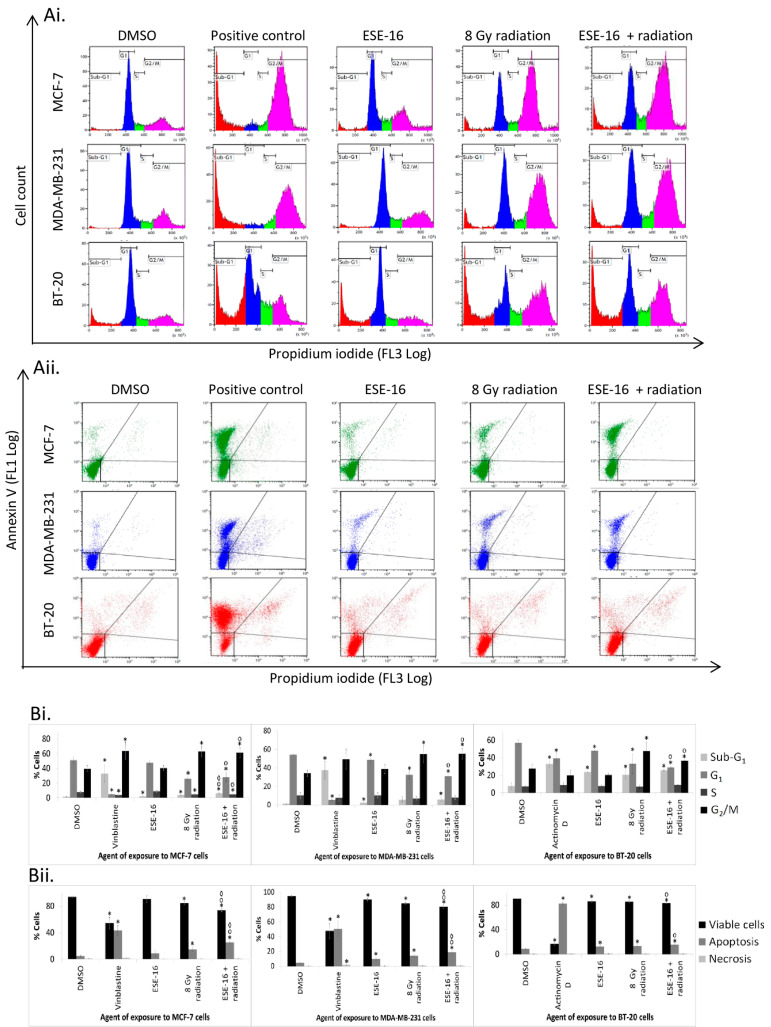
Cell cycle and Annexin V-FITC analysis of cells exposed to the various treatment modalities. (**A**). Cell cycle modifications were visualized by plotting cell counts against propidium iodide (**Ai**), whereas apoptosis was visualized by plotting annexin V-FITC against propidium iodide (bottom left corner: viable cells; top left and right quadrants: apoptotic cells; bottom right quadrant: necrotic cells.) (**Aii**). Cells were exposed to ESE-16 and 8 Gy radiation as experimental controls and compared to the combination treatment. (**B**). Graphical representation of cell cycle analysis (**Bi**) indicated that irradiated cells pre-treated with ESE-16 displayed a significant increase in the G_2_/M phase population with a concurrent decrease in the G_1_ phase population compared to DMSO and compound controls. MCF-7 cells displayed a significant increase of the sub-G_1_ population in the ESE-16/radiation samples when compared to both experimental controls. (**Bii**). Graphical representation of annexin V-FITC quantification in cells exposed to various treatment conditions. BT-20 cells exposed to ESE-16 before 8 Gy radiation displayed a significant increase in the number of apoptotic cells when compared to both experimental controls. MCF-7 and MDA-MB-231 cells exhibited decreased viability and increased apoptotic populations compared to the experimental controls. Averages (n = 3) of viable, apoptotic, and necrotic cells are displayed and STDs are indicated by T-bars. *p* < 0.05 is indicated with * compared to DMSO, ᵒ compared to the ESE-16 drug control, and ^◊^ compared to 8 Gy radiation control.

The effect of ESE-16 (24 h pre-exposure) together with radiation on cell cycle progression was evaluated 24 h after radiation via flow cytometry. BT-20 cells were exposed to actinomycin D, and MCF-7- and MDA-MB-231 cells were exposed to vinblastine as positive method controls for apoptosis, which resulted in significant increases in the amount of cells in the sub-G_1_ phase and apoptotic cell death (Figure 3).

All cells exposed to ESE-16 (as the drug control) (0.157, 0.137, and 0.112 µM, respectively) for 48 h displayed a small but significant increase in the proportion of cells in the apoptotic sub-G_1_ phase (Supplementary data: Table S5). Additionally, ESE-16 exposure significantly decreased the G_1_ population of BT-20- and MDA-MB-231 cells. Exposure to 8 Gy radiation (as the radiation control) significantly increased the number of cells in the G_2_/M phase, while the number of cells in the G_1_ phase was significantly decreased. The sub-G_1_ phase population of BT-20 and MCF-7 cells was slightly increased 24 h after radiation. Compared to the DMSO control, exposure of all three cell lines to the ESE-16/radiation combination significantly increased the sub-G_1_ and G_2_/M populations, whereas the proportion of cells in the G_1_ phase was significantly decreased (Supplementary data: Table S5).

Comparing the cell cycle modifications of BT-20 cells exposed to the combination treatment to those of cells exposed to individual control treatments, the former induced a statistically significant increase in the G_2_/M phase population, with a concurrent decrease in the G_1_ phase population (Figure 3). A decrease in the G_1_ phase population was also observed in MCF-7 cells exposed to the combination treatment, together with a significant increase in the sub-G_1_ and G_2_/M populations compared to cells exposed to the compound only. Compared to cells exposed to the radiation control, the ESE-16/radiation combination treatment resulted in a significant increase in the number of cells in the sub-G_1_ apoptotic phase. Similarly, MDA-MB-231 cells exposed to the combination treatment displayed a significant decrease in the proportion of cells in the G_1_ phase and an increase in the G_2_/M phase population compared to cells exposed to the compound control.

The increased number of cells in the sub-G_1_ phase after exposure to the ESE-16/radiation combination most likely represented an increase in apoptotic cell death. We thus compared the number of apoptotic cells in samples treated with the combination to those exposed to the individual treatments. BT-20 and MDA-MB-231 cells exposed to ESE-16 demonstrated a small but significant decrease in cell viability, together with a small increase in apoptosis (12.56 ± 1.01%) when compared to the vehicle control (Figure 3). ESE-16 exposure did not induce apoptosis in MCF-7 cells at this drug concentration over the given exposure time. Radiation exposure significantly decreased cell viability in all three cell lines (Supplementary data: Table S6). Pre-treatment of MCF-7- and MDA-MB-231 cells with ESE-16 prior to radiation significantly increased apoptosis and decreased cell viability compared to all controls. BT-20 cells exposed to the ESE-16/radiation combination displayed an increased number of apoptotic cells and a decreased viability compared to the DMSO and ESE-16 controls.

These results indicated that pre-treatment of breast cancer cells with ESE-16 may indeed increase the cells’ response to radiation exposure. An augmented effect on the induction of a metaphase block and apoptosis was obtained when the two therapies were combined, with a concomitant decrease in cell viability.

The results of the cell cycle analysis and apoptosis induction should be analyzed taking into account that non-lethal doses of ESE-16 were investigated as a radiosensitizing agent and these studies were aimed more at elucidating the intracellular mechanisms involved. Additionally, these analyses were conducted at an early time point in the response in cell lines that differed substantially in their doubling times.

### 2.4. Long-Term Cellular Proliferation Is Decreased Consequent to Combination Treatment

**Figure 4:**
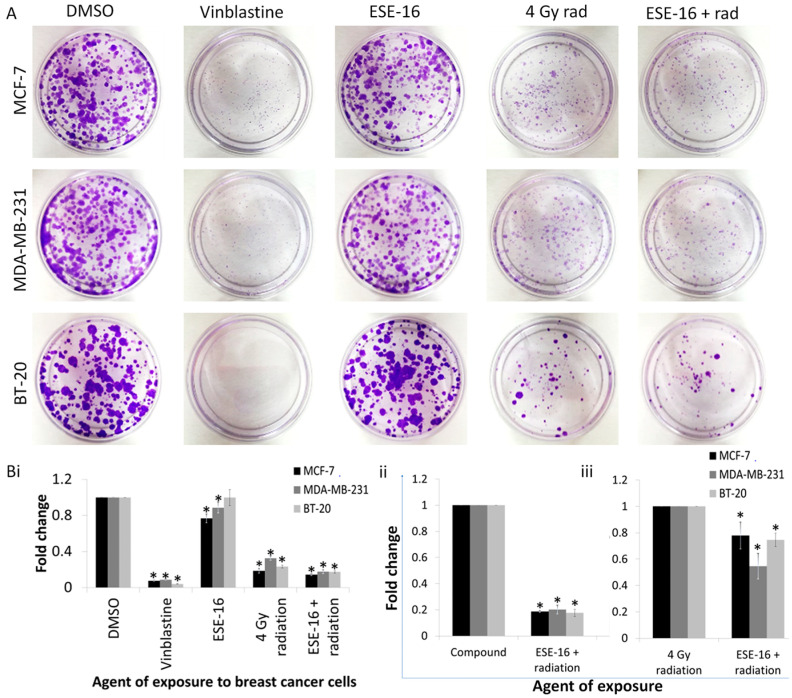
Proliferation of MCF-7, MDA-MB-231, and BT-20 cells 14 days after exposure to the various treatment conditions. (**A**) Representative plates of colonies formed in the various treatment conditions, stained with crystal violet. (**B**) Breast cancer cells exposed to a low dose of ESE-16 24 h prior to 4 Gy radiation showed significantly decreased long-term proliferation when compared to cells exposed to the individual treatments. Bars represent the mean fold changes of three biological repeats and STDs are represented by T-bars. *p* < 0.05 indicated with * compared to DMSO (**Bi**), the compound (**Bii**), or 4 Gy radiation only (**Biii**).

The ability of the breast cancer cells to survive, restore, and proliferate following exposure to the various treatment modalities was quantified using crystal violet staining of the formed colonies 14 days after radiation. Vinblastine, which was used as a positive method control, significantly decreased the ability of the cells to proliferate. MCF-7 and MDA-MB-231 cells exposed to ESE-16 displayed a significant decrease in colony formation (Supplementary data: Table S7). All cells exposed to 4 Gy radiation, as well as the ESE-16/radiation combination, displayed a significant decrease in long-term cellular proliferation compared to the DMSO control (Figure 4). The ESE-16/radiation-treated samples showed a significant decrease in long-term cellular proliferation compared to both the drug and radiation controls in all the cell lines, indicative of an augmented effect of the two treatments.

### 2.5. ESE-16 Pretreatment Affects Radiation-Induced DNA Damage Response and Repair Pathways

To investigate whether pre-treatment with ESE-16 could modify the cellular response to radiation-induced DNA damage, we assessed DNA damage using different approaches at both 2 and 24 h after radiation.

#### 2.5.1. H2AX Phosphorylation Is Sustained in the Combination Treatment

**Figure 5:**
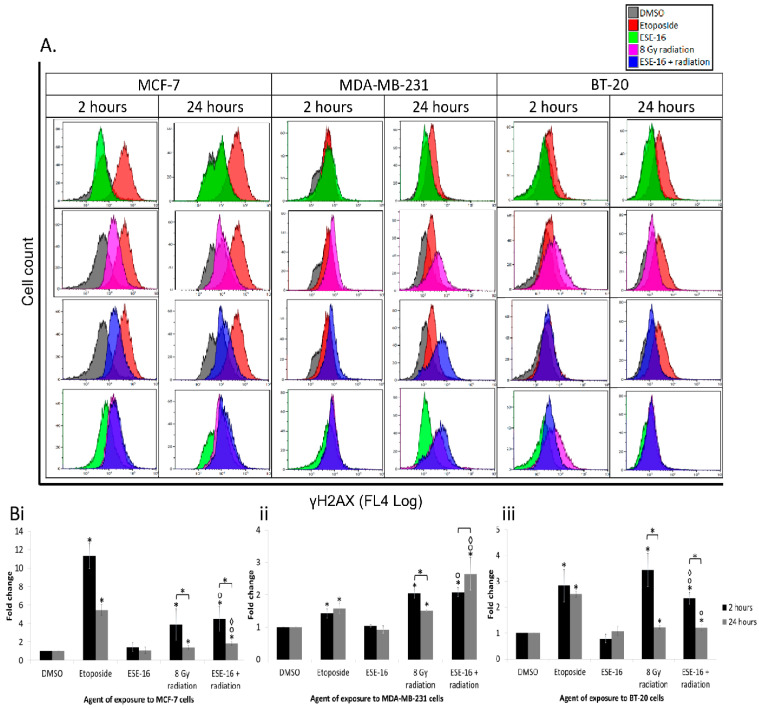
γH2AX detection in breast cancer cells in response to the different treatments. Histograms (**A**) were obtained 2 and 24 h after radiation by plotting cell count against γH2AX level. Overlay histograms displayed a right shift 24 h after radiation in MCF-7 and MDA-BM-231 cells exposed to the combination therapy. (**B**) Graphical representation of the above showed that exposure to 8 Gy radiation resulted in significantly higher γH2AX levels 2 h after radiation, which were partially restored 22 h later. This effect was diminished or lost in the combination-treated MCF-7 (**Bi**) and MDA-MB-231 (**Bii**) cells. Phosphorylation of H2AX was delayed at 2 h in BT-20 cells (**Biii**). Bars represent the mean fold increases of three biological repeats and STDs are represented by T-bars. *p* < 0.05 indicated with *compared to DMSO and 2 h versus (vs.) 24 h, ᵒ compared to ESE-16 drug control, and ^◊^ compared to the 8 Gy radiation control.

Firstly, we measured the phosphorylation of H2AX in response to radiation with and without ESE-16 pre-treatment. All three cell lines exposed to 8 Gy radiation revealed a significant increase in γH2AX levels 2 and 24 h after radiation (Figure 5). However, when compared to the earlier time point (i.e., 2 h after radiation), samples displayed a significant decrease in γH2AX levels 22 h later, indicating the possibility of DNA repair. All cells exposed to ESE-16 24 h before radiation displayed a significant increase in γH2AX levels at 2 h (4.51 ± 1.36-, 2.08 ± 0.14-, and 2.35 ± 0.24-fold increases in MCF-7, MDA-MB-231 and BT-2- cells, respectively) and 24 h (1.83 ± 0.22-, 2.65 ± 0.50-, and 1.21 ± 0.11-fold increases, respectively) after radiation. Similar to the radiation control, ESE-16-pre-treated MCF-7 and BT-20 cells displayed significantly lower levels of γH2AX 24 h after radiation compared to the 2 h time point.

The drug alone had no effect on the phosphorylation of H2AX in any of the cell lines, thereby inferring that the radiation was responsible for the elevated γH2AX levels. Significantly, although the levels of γH2AX in MCF-7 cells decreased at 24 h after an initial spike at 2 h post-radiation, it did so less in the combination-treated cells than in the radiation control. Furthermore, γH2AX levels increased further at 24 h in MDA-MB-231 cells, whilst phosphorylation of H2AX was delayed at the early (2 h) time point in BT-20 cells (0.70 ± 0.12) when compared to the radiation control.

#### 2.5.2. The Number of Micronuclei per Cell Is Increased in Response to the Combination Treatment

**Figure 6:**
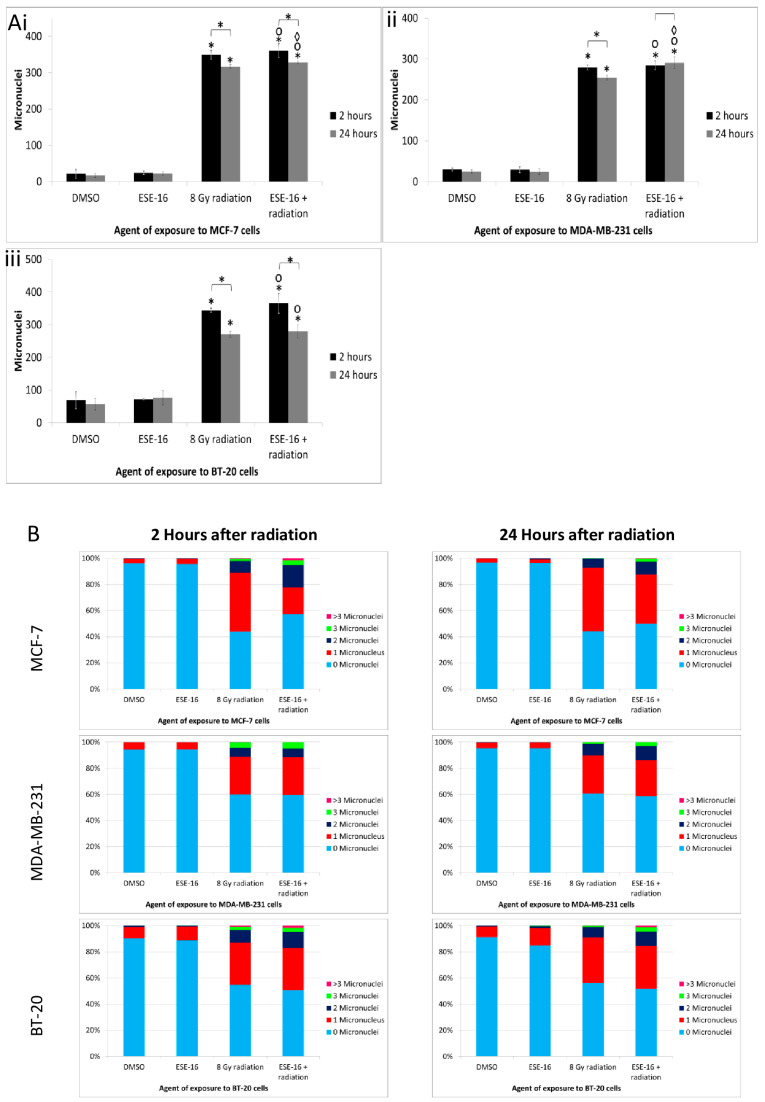
Graphical representation of micronuclei quantification in cells exposed to the various treatment conditions. (**A**) MCF-7 (**i**), MDA-MB-213 (**ii**), and BT-20 cells (**iii**) exposed to ESE-16 displayed no significant increase in the number of micronuclei at both time points. Exposure to 8 Gy radiation resulted in a significant increase in the number of micronuclei 2 h after treatment in all cell lines, which partially decreased over 24 h. MCF-7 and BT-20 cells treated with the combination displayed a similar pattern, although the number of micronuclei remained significantly increased in MCF-7 and MDA-MB-231 cells at 24 h. Bars represent the average number of micronuclei quantified per 500 binucleated cells for three biological repeats with the STDs represented by T-bars. *p* < 0.05 indicated with * compared to DMSO and 2 h vs. 24 h, ᵒ compared to the ESE-16 drug control, and ^◊^ compared to the 8 Gy radiation control. (**B**) Analysis of the distribution of micronuclei within cells exposed to the various treatment conditions showed that there was an increased number of micronuclei per cell in the combination-treated MCF-7 and MDA-MB-231 cells at 24 h compared to their radiation controls. Although the number of cells with DNA damage was not increased in the combination-treated samples, the severity of the DNA damage was amplified, as illustrated by the increased number of micronuclei per cell.

Secondly, we characterized nuclear DNA damage by quantifying the micronuclei formed. Again, no significant increase in DNA damage was observed in ESE-16-exposed cells. Exposure to 8 Gy radiation significantly increased the number of micronuclei 2 h after radiation, with a demonstrable decrease at 24 h (MCF-7: 349 ± 12 and 317 ± 6, respectively; MDA-MB-231: 279 ± 6 and 255 ± 6, respectively; and BT-20 cells: 344 ± 7 and 271 ± 10, respectively) (Figure 6A). ESE-16/radiation-treated MCF-7 and BT-20 cells displayed a decrease in micronuclei numbers 24 h after radiation when compared to their earlier time point. Quantification of micronuclei 2 h after radiation revealed that DNA damage occurred similarly in all cells exposed to radiation and the ESE-16/radiation combination. However, micronuclei formation was significantly higher in the ESE-16/radiation combination-treated MCF-7 and MDA-MB-231 cells 24 h after radiation compared to their relevant radiation controls. Interestingly, the number of cells displaying micronuclei was not increased in the combination-exposed cells, but rather, the number of micronuclei present within a single cell was increased (Figure 6B) (Supplementary data: Table S8). Thus, the number of cells with damaged DNA was similar among cells exposed to the combination treatment and the radiation controls, but the former showed more damage per cell.

#### 2.5.3. Assessment of DNA Damage Response: Ku70 Expression Demonstrated a Delayed Response and a Sustained Increase in the ESE-16/Radiation-Treated Cells

To determine whether the increase in DNA damage was due to an ineffective damage repair mechanism, Ku70 expression was quantified.

**Figure 7:**
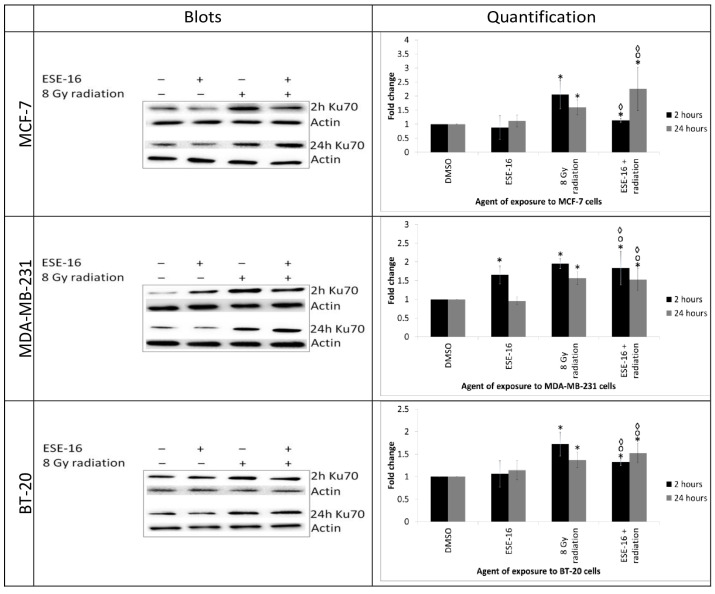
Western blot analysis of Ku70 expression in MCF-7, MDA-MB-231, and BT-20 cells at 2 and 24 h after radiation. Cells exposed to the combination treatment displayed a delayed elevation of Ku70 expression at 2 h, which remained elevated and even increased at 24 h. This was in contrast to the radiation controls, which demonstrated a rapid increase at 2 h that abated at 24 h. Bars represent the mean fold-changes of three biological repeats and STDs are represented by T-bars. *p*-value < 0.05 indicated with * compared to DMSO and 2 h vs. 24 h, ᵒ compared to the ESE-16 drug control, and ^◊^ compared to the 8 Gy radiation control.

A significant increase in Ku70 levels was observed 2 and 24 h after 8 Gy radiation in MCF-7 (2.05 ± 0.51- and 1.60 ± 0.27-fold), MDA-MB-231 (1.95 ± 0.13- and 1.57 ± 0.17-fold), and BT-20 cells (1.72 ± 0.26- and 1.37 ± 0.17-fold) (Figure 7). Elevated Ku70 protein levels were also observed in MDA-MB-231 cells exposed to ESE-16 (1.65 ± 0.24-fold increase) at 2 h, although the rest of the data suggested that the drug did not increase Ku70 expression. The ESE-16/radiation combination treatment significantly amplified Ku70 expression 2 and 24 h after radiation in MCF-7 (1.13 ± 0.07- and 2.26 ± 0.78-fold), MDA-MB-231 (1.83 ± 0.45- and 1.53 ± 0.29-fold), and BT-20 cells (1.32 ± 0.08- and 1.53 ± 0.22-fold). Interestingly, Ku70 expression was decreased at 2 h after radiation in MCF-7 (0.58 ± 0.16), MDA-MB-231 (0.74 ± 0.11), and BT-20 cells (0.78 ± 0.11) exposed to the ESE-16/radiation combination treatment compared to the radiation controls. In contrast, Ku70 expression remained elevated, or even increased, at 24 h in MCF-7 (1.23 ± 0.08-fold), MDA-MB-231 (1.19 ± 0.10-fold), and BT-20 cells (1.11 ± 0.02-fold) in response to the combination treatment.

### 2.6. Radiation-Induced Bystander Effect: DNA Damage and Repair Signaling Molecules Were Transferred via the Conditioned Media

The radiation-induced bystander effect demonstrates the relationship between radiated and non-radiated cells [9]. Non-irradiated cells neighboring treated cells exhibit radiation effects due to the transfer of DNA damage signals [33] As BT-20 cells are most treatment refractive, we used them in this pilot study to investigate a possible bystander effect [34]. The indirect effect of the combination treatment on the activation of the DNA damage response pathway was thus analyzed in cells incubated in the medium collected from cells already treated with ESE-16/radiation and parallel controls.

**Figure 8:**
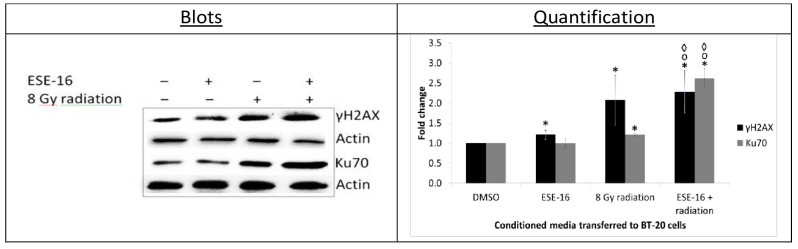
Western blot analysis of γH2AX and Ku70 expression in untreated BT-20 cells exposed for 2 h to conditioned media transferred from cells exposed to the various treatment conditions. Medium transferred from the ESE-16/radiation combination significantly increased the expression of γH2AX and Ku70 in untreated BT-20 cells when compared to those propagated in conditioned media from the radiation and drug controls. Bars indicate the average of three biological repeats, with standard deviations represented by T-bars. Statistically significant differences (*p* < 0.05) are indicated with * compared to DMSO and 2 h vs. 24 h, ᵒ compared to the ESE-16 drug control, and ^◊^ compared to the 8 Gy radiation control.

We observed that the medium from the drug control (which no longer contained ESE-16, see methods) marginally but significantly increased the expression of γH2AX in untreated BT-20 cells (1.21 ± 0.11-fold), while Ku70 expression remained unchanged (Figure 8). A statistically significant increase in γH2AX (2.08 ± 0.63-fold) and Ku70 (1.21 ± 0.03-fold) expression was observed in untreated BT-20 cells propagated in conditioned media obtained from the 8 Gy radiation control. BT-20 cells exposed to the conditioned media obtained from the ESE-16/radiation combination treatment revealed a 2.28 ± 0.53-fold increase in γH2AX levels and a 2.62 ± 0.24-fold increase in Ku70 expression when compared to the vehicle control. Furthermore, these cells displayed a significant increase in γH2AX and Ku70 expression when compared to cells cultured in the conditioned medium of cells exposed to the drug (1.67 ± 0.20- and 2.42 ± 0.67-fold increases, respectively) and 8 Gy radiation (1.34 ± 0.21- and 2.10 ± 0.18-fold increases, respectively) controls.

These results indicated that treated BT-20 cells exerted a bystander effect on non-treated cells via signaling mechanisms and molecules independent of the direct effects of the drug and radiation.

## 3. Discussion

Radiation remains a cornerstone of cancer treatment programs. Although an effective modality, short- and long-term radiation adverse effects may be severe, consequent to non-tumorigenic tissue being harmed in the delivery. This, together with the development of radioresistance, drives research to find a formula in which minimal doses of a chemotherapeutic agent may be administered to sensitize the tumor, which then requires lower radiation doses. In this study, an in silico-designed sulphamoylated 2-ME analogue was investigated as a potential radiosensitizing agent. The primary aim was to elucidate the intracellular signaling mechanisms involved as well as any potential bystander effect on non-irradiated cells.

ESE-16 was cytotoxic to all of the breast cancer cell lines investigated and revealed nanomolar GI_50_ concentrations in MDA-MB-231 (ER/PR-;Her2/Neu-), MCF-7 (ER/PR+; HER2/Neu-) and BT-20 (ER/PR-; HER2/Neu-) cells. Thus, similar to the parent compound 2-ME, ESE-16′s antiproliferative effect appears to be independent of the hormone receptor status of the cells [23,35,36]. Moreover, the compound’s cytotoxicity to MDA-MB-231 and BT-20 cells suggested that further investigation of this drug’s action on triple receptor-negative breast cancers, which are clinically more aggressive and resistant to traditional chemotherapy, may be warranted [34].

Similar to 2-ME, ESE-16 binds to the colchicine-binding site on tubulin and depolymerizes intracellular microtubules in a dose-dependent manner [19,26]. Microtubule abrogation subsequently induces a G_2_/M phase block as a result of activation of the spindle assembly checkpoint [19,28]. Fluorescent microscopy was used in the current study to visualize the microtubule and actin anatomy in MCF-7, MDA-MB-231, and BT-20 cells exposed to ESE-16 for 24 h. Cells displayed depolymerized microtubule networks as well as disrupted actin morphology. Cell migration, as well as cell division, is facilitated by reorganization of the cellular cytoskeleton. Polymerization of the actin skeleton is partially responsible for cell movement and adhesion, and it is regulated by leading and trailing edge regulating proteins trafficked by microtubules [37,38].

Previous data has shown that microtubule abrogation induced by ESE-16 causes a metaphase block with sustained cyclin B levels in HeLa cells treated with IC_50_ concentrations for 24 h [24,26]. Similarly, tetrahydroisoquinoline-based 2-ME analogues caused the accumulation of MDA-MB-231 breast and A549 lung cancer cells in the G_2_/M phase due to depolymerization of the microtubules before inducing apoptosis and autophagy [39]. At doses less than ½ of the IC_50_ values, ESE-16 did not cause a quantifiable metaphase block after 24 h, as observed in this study. However, cell cycle analysis of irradiated cells pre-exposed to ESE-16 displayed a significant increase in the G_2_/M phase population when compared to the compound control. Thus, this metaphase block could be ascribed to the activation of the G_2_/M checkpoint due to DNA damage sustained from the radiation, instead of the effect of the anti-mitotic compound. The initial hypothesis was that exposure to a non-lethal dose of ESE-16 prior to radiation would depress microtubule dynamics, consequently halting the cell cycle at metaphase (the most radiosensitive phase), increasing DNA damage, and resulting in apoptosis [40]. As the drug control did not display a significant mitotic block, other pathways are likely also involved in this treatment interaction.

From the results, the increased sub-G_1_ population of the MCF-7 cells supported the induction of apoptosis, a result substantiated with the annexin V-FITC assay. An alternative possibility is that cell death resulted from perturbations of other microtubule-dependent functions essential for cell survival, such as cytoplasmic-nuclear transport. Low doses of 2-ME and ESE-16 do decrease microtubule dynamics, without initially affecting their anatomy [26]. There is increasing evidence of a G_1_ block and apoptotic signaling via the retinoblastoma (Rb) pathway after treatment with microtubule disrupting agents, as was demonstrated with ESE-16 in MDA-MB-231 and HeLa cells [26,41,42]. Cytotoxicity could also be due to microtubule-independent mechanisms. Reactive oxygen species formation may be integral to this response, as reported by investigations with sister analogue ESE-15-ol in A549 lung cancer cells [43].

The inability of a cell to proliferate and loss of reproductive integrity are the two most common mechanisms of cell death described in radiation biology [44]. Clonogenic studies may be used to evaluate the long-term effectiveness of radiation and/or chemotherapy based on the understanding that senescent cells are unable to multiply due to their incapacity to synthesize DNA and proteins, rendering them non-viable [44,45,46]. In this study, cell populations were stained with crystal violet 14 days after exposure to the ESE-16/4 Gy radiation treatment. A significant decrease in long term cellular proliferation was observed when compared to cells exposed to the experimental controls, indicating an augmented effect of the combination treatment on cellular survival, restoration, and proliferation. These data indicated that the apoptotic signaling demonstrated in the combination-treated MCF-7 and MDA-MB-231 cells had a sustained and long-term consequence. Annexin V-FITC quantification did not reveal an increase in early apoptotic cell death in the more resistant, slowly dividing BT-20 cells line exposed to the ESE-16/radiation therapy. However, the effect of the combination therapy in this cell line could be observed long term in the clonogenic study. Future studies will quantify senescence and the signaling involved in the response to the combination treatment.

DNA double-strand breaks may be sustained in response to radiation exposure [6,47]. γH2AX foci are used as a marker of DNA damage since increased levels correlate with the degree of genotoxic insult [8]. Once the damaged DNA has been identified, recruitment of DNA repair proteins, such as the Ku complex (Ku70/Ku80), is initiated. After repair of the damaged DNA is complete, the Ku complex dissociates from the DNA and the cell cycle commences as the checkpoints are satisfied [8]. DNA damage can also be quantified at the chromosomal level by evaluating the number of micronuclei formed [48]. Damaged chromosomes fail to bind correctly to mitotic spindles during cellular division. This results in the exclusion of either encapsulated chromatid fragments or whole chromosomes from the newly formed daughter-cell nuclei. Micronuclei exhibit a similar morphology as the main nucleus but they are smaller in size [48].

Gorska et al. reported that 2-ME caused DNA damage in an osteosarcoma cell line at physiological and pharmacological concentrations [49]. Exposure to 2-ME for 24 h led to genomic instability within the osteosarcoma cells, resulting in a dose-dependent formation of micronuclei. Furthermore, 10 μM 2-ME increased γH2AX levels in these 143B cells after 2 h. In the present study, however, all cells exposed to the ESE-16 drug control displayed no significant increase in γH2AX levels or micronuclei formation over the 24 h time frame. Additionally, the MCF-7 and BT-20 cells showed no increase in Ku70 expression, indicating that ESE-16 in itself does not appear to be genotoxic. As an outlier of these observations, MDA-MB-231 cells displayed a transient increase in Ku70 expression at 2 h. The exact reason for this increase is unclear. One possibility may be that oxidative estrogen metabolism can generate genotoxic metabolites, such as oxygen free radicals and reactive estrogen quinones, which can interact with DNA and result in unstable DNA adducts [50]. Exposure of hippocampal HT22 cells to 2-ME resulted in genomic instability due to increased nuclear localization of neuronal nitric oxide synthase [50]. Potential genotoxicity of ESE-16 would need further investigation, although ESE-16 exposure did not increase γH2AX foci or micronuclei formation in the studied breast cancer cells, leading to the assumption that any DNA damage induced by this low dose of ESE-16 may be limited.

Since DNA damage was not induced in the drug controls, it is hypothesized that the damage observed after combination treatment stemmed from the radiation, as indicated by increased γH2AX levels and micronuclei formation in all three cell types. Similar results were observed in 2-ME radiosensitized colon carcinoma cells (DLD-1, HCT-8, and HCT-15 cell lines) [51] and radioresistant MCF-7/FIR breast cancer cells [52]. Both studies reported a significant increase in the number of γH2AX foci when irradiated in the presence of 2-ME. Here, DNA repair mechanisms were activated and sustained in MDA-MB-231 and BT-20 cells, as illustrated by the increased Ku70 expression 2 and 24 h after radiation. Ku70 activation in MCF-7 cells appeared delayed compared to the radiation control. Furthermore, attenuation of the DNA damage response over the 24 h time frame was less than in the control, which correlated with the sustained elevation of γH2AX levels in the MDA-MB-231 and MCF-7 cells and the continued elevation of micronuclei numbers. These data suggested that the activation of the repair mechanism may be slightly delayed or that the response may be abrogated. A similar delay in the DNA damage response was reported with an STX3541 (non-steroidal 2-ME analogue) and radiation combination treatment in breast cancer cells, in which the ATM response was depressed in response to increased micronuclei formation [53]. Further investigations are needed to link these findings with the decreased cellular survival quantified in the clonogenic studies. There also appears to be temporal and cell-line specific responses, the details of which remain to be elucidated.

During the cell cycle analysis, BT-20 cells treated with the combination therapy showed only a small increase in the G_2_/M population, along with a larger apoptotic fraction when compared to the other cell lines. Failure to arrest the cell cycle progression appropriately in response to DNA damage may impair the DNA repair response and more rapidly induce apoptosis. Cell cycle signaling molecules need to be investigated in more detail to further expand on this hypothesis. Alternatively, other proteins required for phosphorylation of H2AX in response to dsDNA damage may affect the repair processes. Signaling via the phosphoinositide (PI)-3 kinase pathway is necessary for H2AX phosphorylation, which is affected by ESE-16 [26,54].

Initial postulations included an ineffective shuttling of the DNA repair proteins from the cytoplasm to the nucleus due to the disrupted microtubule and actin dynamics [55]. Nuclear repair mechanisms may also be inhibited by ESE-16 at a transcriptional or posttranscriptional level [56]. Although potentially contributing to the initial observations, ineffective cytoplasmic-nuclear shuttling of DNA repair proteins may not be the exclusive answer to the observation. Signaling molecules released by cells exposed to the combination treatment may be fundamental to activation of the DNA damage response pathway [3,9]. An increase in γH2AX and Ku70 expression was observed in untreated BT-20 cells propagated in the conditioned media obtained from cells exposed to the ESE-16/radiation combination treatment. Due to these results demonstrating a bystander effect, signaling molecules that may extravasate or be part of an intercellular crosstalk are proposed to play a significant role in the activation of the DNA damage response pathway. Future investigations will aim to identify more of the signaling pathways and molecules involved in the bystander effect.

## 4. Materials and Methods

### 4.1. Cell Lines, Culture Method, and Chemicals

The commercial MCF-7 (population doubling time (PDT) of ~38 h), MDA-MB-231 (PDT of ~38 h), and BT-20 (PDT of ~51 h)- tumorigenic human epithelial breast cancer cell lines were acquired from the American Type Culture Collection (ATCC) (Manassas, VA, USA).

MCF-7 and MDA-MB-231 cells were cultured in GIBCO^®^ Dulbecco’s Modified Eagle’s Medium (DMEM), whilst BT-20 cells were cultured in DMEM/GIBCO^®^ HAM F-12 nutrient mixture [50:50) (Thermo Fisher Scientific, Waltham, MA, USA). Growth media were supplemented with 10% heat-inactivated GIBCO^®^ fetal calf serum (FCS), 100 μg/mL streptomycin, penicillin G (100 U/mL), and 250 µg/L fungizone (Thermo Fisher Scientific, Waltham, MA, USA). Cells were maintained in a water-jacketed incubator (Forma Scientific, Marietta, Ohio, USA) at 37 °C under 5% CO_2_. Confluent cells were collected using GIBCO^®^ trypsin-ethylenediaminetetraacetic acid (EDTA) (Thermo Fisher Scientific, Waltham, MA, USA) to detach the cells. Seeding densities are provided in the Supplementary data, Section S1.

The non-commercially available sulphamoylated 2-ME analogue, ESE-16, was synthesized by iThemba (Pty) Ltd. Pharmaceuticals (Modderfontein, Gauteng, South Africa). All other chemicals not specifically mentioned were of analytical grade and were purchased from Sigma-Aldrich (St. Louis, MO, USA or Saint-Quentin-Fallavier, France). 

Adherent MCF-7, MDA-MB-231, and BT-20 cells were exposed to 0.157, 0.137, and 0.112 µM of ESE-16, respectively, for 24 h. The lowest dose that significantly increased apoptosis was determined by a compound dose-response curve (Supplementary data: Sections S2 and S3). Treated cells were radiated with 8 Gy radiation (as determined by a radiation dose-response curve (Supplementary data: Sections S4 and S5). Analysis was conducted 2 and 24 h after radiation. The radiation-induced bystander effect was investigated by incubating non-radiated BT-20 cells with conditioned medium collected from BT-20 cells exposed to the combination treatment. The conditioned medium was created by exposing BT-20 cells to the drug for 24 h, which was removed prior to radiation with 8 Gy. After radiation, the cells were incubated for 2 h. These conditioned media were then transferred onto 80% confluent BT-20 cells for 2 h prior to DNA damage and repair analysis.

Samples were radiated using a BloodXrad 13–69 (Le-Plessis-Pâté, France) with a dose rate of 7 Gy/min or on a Siemens Oncor 6 MV linear accelerator with a dose rate of 1 centigray (cGy)/ monitor unit (MU) using a 10 × 10 cm field with an appropriate bolus to ensure that each sample received the full radiation dose.

### 4.2. Experimental and Method Controls

Cells propagated in growth medium only served as negative controls. A DMSO vehicle control consisted of the same amount of DMSO (*v*/*v*%) used as the excipient (not exceeding 0.5%). Each experiment employed an appropriate positive method control. The experimental controls encompassed cells exposed to radiation only (terminated 24 h after radiation) or compound only (terminated 48 h after exposure).

### 4.3. Determination of ESE-16 Cytotoxicity

The GI_50_ of ESE-16 was determined in MCF-7, MDA-MB-231, and BT-20 cells by spectrophotometric quantification of formazan crystals using 3-(4,5-dimethylthiazol-2-yl)-2,5-diphenyltetrazolium bromide (MTT) (Sigma Aldrich, St. Louis, MO, USA). Viable cells were exposed to a dilution series of ESE-16 for 48 h. Cells were incubated in 5 mg/mL MTT (4 h at 37 °C) protected from light. MTT solvent (isopropanol, 0.1 N HCl, and 10% Triton^®^ X-100) was added and plates were incubated overnight at room temperature (RT) in the dark. The absorbance was read at 570 nm using an ELx800 Universal Microplate Reader (Bio-Tek Instruments Inc., Winooski, VT, USA). Data of three independent biological repeats (n = 4) were analyzed. Cell viability was calculated relative to the DMSO vehicle control and expressed as percentage cell growth. The GI_50_ (drug concentration causing 50% inhibition of cell growth) values were calculated from the dose response curves.

Cells were further exposed to a dilution series of the compound (GI_50_, ¾ GI_50_, ½ GI_50_, ¼ GI_50_, and ⅙ GI_50_) as well as a radiation dose-response curve (2, 4, 6, 8, and 10 Gy) to determine the lowest compound and radiation doses that significantly induced apoptosis as monotherapies (as determined by flow cytometry using Annexin V (BioLegend, San Diego, CA, USA) and cell cycle analysis (Supplementary data; Figures S1–S8)). These concentrations were used for sensitization studies.

### 4.4. Immunofluorescence Microscopy of the Intracellular Cytoskeletal Anatomy

BT-20, MCF-7, and MDA-MB-231 cells were seeded on 12 mm round sterilized coverslips and allowed to attach for 48 h before exposure to ESE-16 for 24 h (at GI_50_ concentrations). Cells were fixed in 3.7% formaldehyde/PBS for 30 min at 37 °C. Cells were permeabilized with 0.2% Triton^®^ X-100/PBS (EUROMEDEX, Mundolsheim, France) for 30 min at RT. Cells were incubated with primary antibody (primary monoclonal mouse α-tubulin antibody (α3A1) (1:4000; L. Lafanechère, Grenoble, France [57]) and Rhodamine Phalloidin (1:1000; Invitrogen, Carlsbad, CA, USA) in 0.1% PBS-Tween^®^-20 with 2% bovine serum albumin (BSA) for 30 min at RT. Samples were rinsed with PBS-Tween^®^-20 (0.1%) before incubation for 30 min with secondary antibody (Alexa Fluor^®^ 488 goat anti-mouse immunoglobulin G (IgG) (Jackson ImmunoResearch Laboratories, West Grove, PA, USA) at RT. Coverslips were mounted on microscope slides with ProLong^®^ Diamond Antifade Mountant with DAPI (Invitrogen, Carlsbad, CA, USA). A Zeiss ApoTome microscope with Axiovision software was used to view the slides (63× oil objective) and images were captured using an AxioCam MRm camera (Zeiss, Oberkochen, Germany).

### 4.5. Flow Cytometric Quantification of Apoptosis Using Annexin V-Fluorescein Isothiocyanate (FITC) Detection

The FITC Annexin V apoptosis detection kit from BioLegend (San Diego, CA, USA) was used to detect the externalization of phosphatidylserine in apoptotic cells. Treated cells were resuspended in Annexin V binding buffer (0.25–0.5 × 10^7^ cells/mL). Cell suspension (100 µL) was transferred to test tubes to which Annexin V-FITC (5 µL) and 10 µL of propidium iodide (PI) were added (15 min, RT). Annexin V binding buffer was added and cells were analyzed using an FC500 system flow cytometer (Beckman Coulter, Brea, CA, USA). The fluorescence of the FL1 channel (emissions at 515–545 nm) for the detection of FITC was plotted against the emission from the FL3 channel (emissions at 600 nm) for the detection of PI.

### 4.6. Cell Cycle Analysis Using Flow Cytometry

Treated cells were resuspended in 200 µL ice-cold PBS/0.1% FCS. Ice-cold 70% ethanol was added and cells were incubated for 24 h at 4 °C. Pelleted cells were resuspended in PBS containing PI (40 µg/mL), Triton^®^ X-100 (0.1%), and ribonucleic acid (RNA)se A (100 µg/mL) for 40 min at 37 °C. PI fluorescence was detected on the FL3 channel (excitation/emission at 535/617 nm) using an FC500 system flow cytometer (Beckman Coulter, Brea, CA, USA).

### 4.7. Cell Survival Assay and Long-Term Cellular Proliferation Analysis Using Clonogenic Studies

Cellular survival and long-term proliferation following exposure to the different treatment modalities were assessed by quantifying the colonies formed 14 days after radiation. Due to cells being seeded at very low densities for this experiment, the dose of radiation was decreased to 4 Gy and kept constant throughout this experiment. Treated MDA-MB-231 and MCF-7 cells were seeded at 8 × 10^4^ cells per 30 mm petri dish in DMEM, and BT-20 cells were seeded at 10 × 10^4^ and at 8 × 10^4^ cells per 30 mm petri dish in DMEM/GIBCO^®^ HAM F-12 nutrient mixture (50:50). Cells were allowed to form colonies for 14 days, with a media change every 3rd day. Colonies were stained with crystal violet (0.05% crystal violet, 1% formaldehyde, 1× PBS, and 1% methanol) for 1 h at RT. Plates were rinsed with distilled water and dried. The crystal violet dye was solubilized with 0.2% Triton^®^ X-100 (30 min, RT). The absorbance was read at 570 nm using a FLUOstar OPTIMA Microplate Reader (BMG LabTech, Ortenberg, Germany). Data from three independent biological repeats (n = 3) were analyzed for each cell line.

### 4.8. Quantification of Phosphorylated H2AX via Flow Cytometry

The FlowCellect™ DNA Damage Histone H2AX Dual Detection Kit (Merck Millipore, Munich, Germany) was used to quantify the phosphorylation status of H2AX according to the manufacturer’s protocol. Briefly, trypsinized cells were washed in 1× wash buffer and fixed with a mixture of fixation buffer and 1x wash buffer (1:1) (10 min on ice). After sedimentation, cells were resuspended in 1x assay buffer and centrifuged again at 300 × *g* for 3 min at 4 °C. Cells were resuspended in ice-cold permeabilization buffer (20 min on ice). Cells were incubated in assay buffer containing anti-phospho-histone H2A.X-peridinin chlorophyll protein complex (PerCP) (monoclonal antibody) (30 min, RT). Fluorescence on the FL4 channel (excitation/emission at 477/678 nm) was measured with an FC500 system flow cytometer (Beckman Coulter, Brea, CA, USA).

### 4.9. Quantification of Micronuclei As an Indication of DNA Damage

The number of micronuclei was quantified in binucleated cells. MCF-7, MDA-MB-231, and BT-20 cells were exposed to cytochalasin B in fresh medium 2 and 24 h after radiation to prevent cytokinesis. Cytochalasin B exposure was timed for one and a half times the doubling time of each cell line before experimental termination.

Pre-warmed hypotonic potassium chloride solution (0.14 M KCl) was added to the treated pelleted cells (with agitation), which were incubated (5 min, RT). Sedimented cells were incubated in fixative I (methanol: 0.9% NaCl: acetic acid [*v*:*v*:*v*; 12:13:3]) at RT for 5 min. After centrifugation, cells were incubated in fixative II (methanol: acetic acid [*v*:*v*; 4:1]) (RT, 5 min). Three drops of each pelleted sample were placed on degreased, dry microscope slides. Slides were dried overnight and stained with 10% Giemsa. Samples were visualized under a light microscope followed by blind scoring of micronuclei in 500 binucleated cells per repeat.

### 4.10. Western Blot Analysis

A standard western blot protocol was used. Treated cells were lysed with ice-cold radioimmunoprecipitation assay (RIPA) buffer (150 mM NaCl, 10 mM Tris-HCl (pH 7.4), 0.1% SDS, 0.5% sodium deoxycholate, 1 mM EDTA, 1 mM EGTA, 1× protease inhibitor, and 1× phosphatase inhibitor cocktails 2 and 3) for 5 min on ice. Lysates were collected via scraping followed by centrifugation (21,130 × *g* at 4 °C for 30 min) (Eppendorf^®^ Centrifuge 5424 R, Hamburg, Germany). The Bradford protein assay (Bio-Rad Laboratories Inc., Hercules, CA, USA) was used to determine the protein concentration of each sample by adding 2–3 µL of the cytosolic extract to 500 µL ddH_2_O in a BioSigma BSA002 Cuvette (Veneto, Italy), followed by the addition of 500 µL Bradford reagent. The absorbance was measured at 595 nm (BioPhotometer Plus, Eppendorf^®^, Hamburg, Germany). The protein concentration of the samples was calculated using a BSA standard curve.

Cellular protein (20 µg) of each sample was denatured at 96 °C for 5 min in Laemmli buffer (Eppendorf^®^ Thermomixer Compact, Eppendorf^®^, Hamburg, Germany). Proteins, together with the Precision Plus protein dual color marker (Bio-Rad Laboratories Inc., Hercules, CA, USA) were separated via sodium dodecyl sulfate-polyacrylamide gel electrophoresis (SDS-PAGE) by applying 80 volts for 3 h in 1× Tris-Glycine-SDS migration buffer (EUROMEDEX, Mundolsheim, France) using a Mini-PROTEAN^®^ Tetra Vertical Electrophoresis Cell (Bio-Rad Laboratories Inc., Hercules, CA, USA). Proteins were transferred to a Trans-Blot^®^ Turbo™ Midi-size polyvinylidene difluoride (PVDF) membrane (Bio-Rad Laboratories Inc., Hercules, CA, USA), which was activated in 100% ethanol, in transfer buffer (25 mM Tris-HCl, 190 mM glycine, 20% ethanol, pH 8.3). Wet transfer was achieved by applying 100 V to the sandwiched membrane for 90 min. Membranes were incubated in 5% BSA 0.1% Tris-buffered saline (TBS)-Tween^®^-20 (TBST) for 90 min before overnight incubation (4 °C) in the primary antibody cocktail (mouse anti-phospho-histone H2A.X (Ser139) monoclonal antibody (Merck Millipore, Cat. No. 05-636) or mouse anti-Ku70 monoclonal antibody (Santa Cruz Biotechnology Inc., Cat. No. sc-56129) in 5% BSA TBST. Membranes were washed with TBST before a 1 h incubation with the secondary antibody (donkey anti-mouse horseradish peroxidase (HRP)-conjugated polyclonal antibody; Jackson ImmunoResearch, Cat. No. 715-035-150) in 2% BSA TBST (RT). Membranes were washed three times with TBST. Proteins were visualized using ChemiDoc MP (Bio-Rad Laboratories Inc., Hercules, CA, USA) after activating HRP activity with Clarity™ Western ECL Substrate (Bio-Rad Laboratories Inc., Hercules, CA, USA). Protein bands were analyzed following standardization to β-actin produced in rabbit (Cell Signalling Technology, Cat. No. 4967) using Image Lab version 5.2.1 software (Bio-Rad Laboratories Inc., Hercules, CA, USA).

### 4.11. Statistical Analysis

Quantitative data were obtained from three independent biological repeats. Flow cytometric data of at least 10,000 cells were obtained for each repeat. Kaluza Analysis version 1.5 software (Beckman Coulter, Brea, CA, USA) was used to analyze all flow cytometry data. Western blot band densitometry was determined using Image Lab version 5.2.1 software (Bio-Rad Laboratories Inc., Hercules, CA, USA). Quantification of western blot bands was determined following standardization, using bands obtained from actin staining as a housekeeping protein. Blind scoring of micronuclei was performed in triplicate (500 binucleated cells) for each treatment condition and all relevant controls. Analysis of variance (ANOVA)-single factor model followed by a two-tailed Student’s *t*-test was used for statistical analysis. A *p*-value < 0.05 was regarded as statistically significant. The combined treatment conditions were compared to the DMSO vehicle control, as well as to the parallel individual drug and radiation treatment samples as experimental controls.

## 5. Conclusions

Overall, the results indicated that pre-treatment of breast cancer cells with a low dose of the microtubule-regulating drug ESE-16 sensitized the cells to radiation in vitro. Alterations in the microtubule network may increase the time spent in metaphase (metaphase block), the most radiosensitive phase of the cell cycle. Other potential mechanisms, such as the enhanced production of reactive oxygen species by inhibition of the electron transport chain complex 1 [58], prevention of G_1_/S progression [59,60,61], as well as ineffective shuttling of nuclear repair machinery, may also play a potential role. Overall, ESE-16 treatment augmented the DNA damage induced by radiation, while delaying the DNA repair response, thereby decreasing the long-term survival of the treated cells. Small molecules from the irradiated cells acted in a paracrine manner on unexposed cells, indicating that a bystander effect can be quantified. The exact ramifications of this phenomenon, as well as the signaling molecules and pathways involved, will be the topic of future research.

## Data Availability

Not applicable.

## Supplementary Materials

The supporting information can be downloaded at: https://www.mdpi.com/article/10.3390/ijms24043592/s1.

## References

[1] Durante M., Loeffler J.S. (2010). Charged particles in radiation oncology. Nat. Rev. Clin. Oncol..

[2] Peters L.J., Withers H.R., Thames H.D., Fletcher G.H. (1982). Tumor radioresistance in clinical radiotherapy. Int. J. Radiat. Oncol. Biol. Phys..

[3] West C.M., Davidson S.E., Hendry J.H., Hunter R.D. (1991). Prediction of cervical carcinoma response to radiotherapy. Lancet.

[4] Maier P., Hartmann L., Wenz F., Herskind C. (2016). Cellular Pathways in Response to Ionizing Radiation and Their Targetability for Tumor Radiosensitization. Int. J. Mol. Sci..

[5] Mao Z., Bozzella M., Seluanov A., Gorbunova V. (2008). Comparison of nonhomologous end joining and homologous recombination in human cells. DNA Repair.

[6] Helena J.M., Joubert A.M., Grobbelaar S., Nolte E.M., Nel M., Pepper M.S., Coetzee M., Mercier A.E. (2018). Deoxyribonucleic Acid Damage and Repair: Capitalizing on Our Understanding of the Mechanisms of Maintaining Genomic Integrity for Therapeutic Purposes. Int. J. Mol. Sci..

[7] Enriquez-Rios V., Dumitrache L.C., Downing S.M., Li Y., Brown E.J., Russell H.R., McKinnon P.J. (2017). DNA-PKcs, ATM, and ATR Interplay Maintains Genome Integrity during Neurogenesis. J. Neurosci..

[8] Meyer B., Voss K.O., Tobias F., Jakob B., Durante M., Taucher-Scholz G. (2013). Clustered DNA damage induces pan-nuclear H2AX phosphorylation mediated by ATM and DNA-PK. Nucleic Acids Res..

[9] Najafi M., Fardid R., Hadadi G., Fardid M. (2014). The mechanisms of radiation-induced bystander effect. J. Biomed. Phys. Eng..

[10] Yang H., Asaad N., Held K.D. (2005). Medium-mediated intercellular communication is involved in bystander responses of X-ray-irradiated normal human fibroblasts. Oncogene.

[11] Xu S., Wang J., Ding N., Hu W., Zhang X., Wang B., Hua J., Wei W., Zhu Q. (2015). Exosome-mediated microRNA transfer plays a role in radiation-induced bystander effect. RNA Biol..

[12] Oyamada M., Oyamada Y., Takamatsu T. (2005). Regulation of connexin expression. Biochim. Biophys. Acta.

[13] Zhu B.T., Conney A.H. (1998). Functional role of estrogen metabolism in target cells: Review and perspectives. Carcinogenesis.

[14] Sawicka E., Saczko J., Roik J., Kulbacka J., Piwowar A. (2020). Effect of Interaction between 17β-Estradiol, 2-Methoxyestradiol and 16α-Hydroxyestrone with Chromium (VI) on Ovary Cancer Line SKOV-3: Preliminary Study. Molecules.

[15] Cavalieri E., Rogan E. (2021). The 3,4-Quinones of Estrone and Estradiol Are the Initiators of Cancer whereas Resveratrol and N-acetylcysteine Are the Preventers. Int. J. Mol. Sci..

[16] Sweeney C., Liu G., Yiannoutsos C., Kolesar J., Horvath D., Staab M.J., Fife K., Armstrong V., Treston A., Sidor C. (2005). A phase II multicenter, randomized, double-blind, safety trial assessing the pharmacokinetics, pharmacodynamics, and efficacy of oral 2-methoxyestradiol capsules in hormone-refractory prostate cancer. Clin. Cancer Res..

[17] Kulke M.H., Chan J.A., Meyerhardt J.A., Zhu A.X., Abrams T.A., Blaszkowsky L.S., Regan E., Sidor C., Fuchs C.S. (2011). A prospective phase II study of 2-methoxyestradiol administered in combination with bevacizumab in patients with metastatic carcinoid tumors. Cancer Chemother. Pharmacol..

[18] Matei D., Schilder J., Sutton G., Perkins S., Breen T., Quon C., Sidor C. (2009). Activity of 2 methoxyestradiol (Panzem NCD) in advanced, platinum-resistant ovarian cancer and primary peritoneal carcinomatosis: A Hoosier Oncology Group trial. Gynecol. Oncol..

[19] Mooberry S.L. (2003). Mechanism of action of 2-methoxyestradiol: New developments. Drug Resist. Update.

[20] Tevaarwerk A.J., Holen K.D., Alberti D.B., Sidor C., Arnott J., Quon C., Wilding G., Liu G. (2009). Phase I trial of 2-methoxyestradiol NanoCrystal dispersion in advanced solid malignancies. Clin. Cancer Res..

[21] Stander A., Joubert F., Joubert A. (2011). Docking, synthesis, and in vitro evaluation of antimitotic estrone analogs. Chem. Biol. Drug Des..

[22] Mothibeli K.T., Mercier A.E., Cromarty A.D., Rheeder M., Naidoo V., Olorunju S.A.S., Joubert A.M. (2018). Confirming oral bioavailability of novel oestradiol analogues by liquid chromatography-tandem mass spectrometry in a murine model. Biomed. Res..

[23] Theron A., Prudent R., Nolte E., van den Bout I., Punchoo R., Marais S., du Toit P., Hlophe Y., van Papendorp D., Lafanechère L. (2015). Novel in silico-designed estradiol analogues are cytotoxic to a multidrug-resistant cell line at nanomolar concentrations. Cancer Chemother. Pharmacol..

[24] Theron A., Nolte E., Lafanechere L., Joubert A. (2013). Molecular crosstalk between apoptosis and autophagy induced by a novel 2-methoxyestradiol analogue in cervical adenocarcinoma cells. Cancer Cell Int..

[25] Visagie M.H., Theron A.E., Mqoco T., Vieira W.A., Prudent R., Martinez A., Lafanechère L., Joubert A.M. (2013). Sulphamoylated 2-methoxyestradiol analogues induce apoptosis in adenocarcinoma cell lines. PLoS ONE.

[26] Mercier A.E., Prudent R., Pepper M.S., De Koning L., Nolte E., Peronne L., Nel M., Lafanechère L., Joubert A.M. (2021). Characterization of signalling pathways that link apoptosis and autophagy to cell death induced by estrone Analogues which reversibly depolymerize microtubules. Molecules.

[27] Verwey M., Nolte E.M., Joubert A.M., Theron A.E. (2016). Autophagy induced by a sulphamoylated estrone analogue contributes to its cytotoxic effect on breast cancer cells. Cancer Cell Int..

[28] van Vuuren R.J., Visagie M.H., Theron A.E., Joubert A.M. (2015). Antimitotic drugs in the treatment of cancer. Cancer Chemother. Pharmacol..

[29] Pawlik T.M., Keyomarsi K. (2004). Role of cell cycle in mediating sensitivity to radiotherapy. Int. J. Radiat. Oncol. Biol. Phys..

[30] Masunaga S.I., Ono K., Suzuki M., Nishimura Y., Kinashi Y., Takagaki M., Hori H., Nagasawa H., Uto Y., Tsuchiya I. (2001). Radiosensitization effect by combination with paclitaxel in vivo, including the effect on intratumor quiescent cells. Int. J. Radiat. Oncol. Biol. Phys..

[31] Stander X.X., Stander B.A., Joubert A.M. (2011). In vitro effects of an in silico-modelled 17β-estradiol derivative in combination with dichloroacetic acid on MCF-7 and MCF-12A cells. Cell Prolif..

[32] Helena J., Joubert A., Mabeta P., Coetzee M., Lakier R., Mercier A. (2021). Intracellular Signaling Responses Induced by Radiation within an In Vitro Bone Metastasis Model after Pre-Treatment with an Estrone Analogue. Cells.

[33] Nagasawa H., Little J.B. (1992). Induction of sister chromatid exchanges by extremely low doses of alpha-particles. Cancer Res..

[34] Yao H., He G., Yan S., Chen C., Song L., Rosol T.J., Deng X. (2017). Triple-negative breast cancer: Is there a treatment on the horizon?. Oncotarget.

[35] LaVallee T.M., Zhan X.H., Herbstritt C.J., Kough E.C., Green S.J., Pribluda V.S. (2002). 2-Methoxyestradiol inhibits proliferation and induces apoptosis independently of estrogen receptors α and β. Cancer Res..

[36] Lee S.T., Lee J.Y., Han C.R., Kim Y.H., Taub D., Kim Y.H. (2015). Dependency of 2-methoxyestradiol-induced mitochondrial apoptosis on mitotic spindle network impairment and prometaphase arrest in human Jurkat T cells. Biochem. Pharmacol..

[37] Raftopoulou M., Hall A. (2004). Cell migration: Rho GTPases lead the way. Dev. Biol..

[38] Small J.V., Kaverina I. (2003). Microtubules meet substrate adhesions to arrange cell polarity. Curr. Opin. Cell Biol..

[39] Nel M., Joubert A.M., Dohle W., Potter B.V.L., Theron A.E. (2018). Modes of cell death induced by tetrahydroisoquinoline-based analogs in MDA-MB-231 breast and A549 lung cancer cell lines. Drug Des. Devel. Ther..

[40] Murr R., Vaissière T., Sawan C., Shukla V., Herceg Z. (2007). Orchestration of chromatin-based processes: Mind the TRRAP. Oncogene.

[41] Biasoli D., Kahn S.A., Cornélio T.A., Furtado M., Campanati L., Chneiweiss H., Moura-Neto V., Borges H.L. (2013). Retinoblastoma protein regulates the crosstalk between autophagy and apoptosis, and favors glioblastoma resistance to etoposide. Cell Death Dis..

[42] Bosco E.E., Wang Y., Xu H., Zilfou J.T., Knudsen K.E., Aronow B.J., Lowe S.W., Knudsen E.S. (2007). The retinoblastoma tumor suppressor modifies the therapeutic response of breast cancer. J. Clin. Investig..

[43] Nolte E., Joubert A., Lakier R., van Rensburg A., Mercier A. (2018). Exposure of breast and lung cancer cells to a novel estrone analog prior to radiation enhances Bcl-2-mediated cell death. Int. J. Mol. Sci..

[44] Munshi A., Hobbs M., Meyn R.E. (2005). Clonogenic cell survival assay. Methods Mol. Med..

[45] Rafehi H., Orlowski C., Georgiadis G.T., Ververis K., El-Osta A., Karagiannis T.C. (2011). Clonogenic assay: Adherent cells. J. Vis. Exp..

[46] Puck T.T., Marcus P.I. (1956). Action of x-rays on mammalian cells. J. Exp. Med..

[47] Barnard S., Bouffler S., Rothkamm K. (2013). The shape of the radiation dose response for DNA double-strand break induction and repair. Genome Integr..

[48] Luzhna L., Kathiria P., Kovalchuk O. (2013). Micronuclei in genotoxicity assessment: From genetics to epigenetics and beyond. Front. Genet..

[49] Gorska M., Wyszkowska R.M., Kuban-Jankowska A., Wozniak M. (2016). Impact of Apparent Antagonism of Estrogen Receptor β by Fulvestrant on Anticancer Activity of 2-Methoxyestradiol. Anticancer Res..

[50] Gorska M., Zmijewski M.A., Kuban-Jankowska A., Wnuk M., Rzeszutek I., Wozniak M. (2016). Neuronal Nitric Oxide Synthase-Mediated Genotoxicity of 2-Methoxyestradiol in Hippocampal HT22 Cell Line. Mol. Neurobiol..

[51] Zou H., Adachi M., Imai K., Hareyama M., Yoshioka K., Zhao S., Shinomura Y. (2006). 2-methoxyestradiol, an endogenous mammalian metabolite, radiosensitizes colon carcinoma cells through c-Jun NH2-terminal kinase activation. Clin. Cancer Res..

[52] Salama S., Diaz-Arrastia C., Patel D., Botting S., Hatch S. (2011). 2-Methoxyestradiol, an endogenous estrogen metabolite, sensitizes radioresistant MCF-7/FIR breast cancer cells through multiple mechanisms. Int. J. Radiat. Oncol. Biol. Phys..

[53] Hargrave S.D., Joubert A.M., Potter B.V.L., Dohle W., Marais S., Mercier A.E. (2022). Cell Fate following Irradiation of MDA-MB-231 and MCF-7 Breast Cancer Cells Pre-Exposed to the Tetrahydroisoquinoline Sulfamate Microtubule Disruptor STX3451. Molecules.

[54] Paull T.T., Rogakou E.P., Yamazaki V., Kirchgessner C.U., Gellert M., Bonner W.M. (2000). A critical role for histone H2AX in recruitment of repair factors to nuclear foci after DNA damage. Curr. Biol..

[55] Dittmann K., Mayer C., Fehrenbacher B., Schaller M., Raju U., Milas L., Chen D.J., Kehlbach R., Rodemann H.P. (2005). Radiation-induced epidermal growth factor receptor nuclear import is linked to activation of DNA-dependent protein kinase. J. Biol. Chem..

[56] Chang L., Graham P.H., Hao J., Ni J., Bucci J., Cozzi P.J., Kearsley J.H., Li Y. (2014). PI3K/Akt/mTOR pathway inhibitors enhance radiosensitivity in radioresistant prostate cancer cells through inducing apoptosis, reducing autophagy, suppressing NHEJ and HR repair pathways. Cell Death Dis..

[57] Paturle-Lafanechere L., Edde B., Denoulet P., Van Dorsselaer A., Mazarguil H., Le Caer J.P., Wehland J., Job D. (1991). Characterization of a major brain tubulin variant which cannot be tyrosinated. Biochemistry.

[58] Hagen T., D’Amico G., Quintero M., Palacios-Callender M., Hollis V., Lam F., Moncada S. (2004). Inhibition of mitochondrial respiration by the anticancer agent 2-methoxyestradiol. Biochem. Biophys. Res. Commun..

[59] Khan S.H., Wahl G.M. (1998). p53 and pRb prevent rereplication in response to microtubule inhibitors by mediating a reversible G1 arrest. Cancer Res..

[60] Mikule K., Delaval B., Kaldis P., Jurcyzk A., Hergert P., Doxsey S. (2007). Loss of centrosome integrity induces p38-p53-p21-dependent G1-S arrest. Nat. Cell Biol..

[61] Ikui A.E., Yang C.P., Matsumoto T., Horwitz S.B. (2005). Low concentrations of taxol cause mitotic delay followed by premature dissociation of p55CDC from Mad2 and BubR1 and abrogation of the spindle checkpoint, leading to aneuploidy. Cell Cycle.

