# Supplementary material for: Radiosensitization of Breast Cancer Cells with a 2-Methoxyestradiol Analogue Affects DNA Damage and Repair Signaling In Vitro
Source: Int J Mol Sci. 2023 Feb 10;24(4):3592. doi: 10.3390/ijms24043592 (PMC9965329; doi:10.3390/ijms24043592)
Supplement: Supplementary file 1 [file ijms-24-03592-s001.zip › ijms-2132226-supplementary.pdf]

Supplementary information and data

Nolte *et al.* IJMS 2023

S1. Seeding concentration of cells for each experiment

MCF-7- and MDA-MB-231 cells were seeded at a density of $5X10^3$ cells/96-well plate for cytotoxicity studies, $4X10^4$ cells/24-well plate for fluorescent microscopy, $8X10^4$ cells/6-well plate for long term cellular proliferation studies (clonogenic studies) and $5X10^5$/25 cm^2 flask for Western blot, micronuclei and flow cytometry experiments. BT-20 cells were seeded at a density of $7X10^3$ cells/96-well plate for cytotoxicity studies, $6X10^4$ cells/24-well plate for fluorescent microscopy, $1X10^5$ cells/6-well plate for long term cellular proliferation studies (clonogenic studies) and $1X10^6$/25 cm^2 flask for western blot, micronuclei and flow cytometry experiments.

S2. Compound dose-response curve: Flow cytometric quantification of apoptosis induction

Breast cancer cells were exposed to the lowest compound dose that significantly increased apoptosis to sensitize the cells to radiation. These doses were determined by exposing the cells to a compound dose-response curve (GI_{50}, ¾ GI_{50}, ½ GI_{50}, ¼ GI_{50} or ⅙ GI_{50}) followed by quantification of apoptosis 48 hours after exposure via flow cytometry employing annexin V.

Breast cancer cells were exposed to DMSO as a vehicle control and actinomycin D (BT-20 cells) or vinblastine (MCF-7- and MDA-MB-231 cells) as positive controls for apoptosis. No statistically significant differences were observed in cell viability or apoptosis in the DMSO samples compared to cells propagated in medium only. Compared to the vehicle control cells exposed to the positive apoptotic controls displayed a decrease in cell viability in addition to increased apoptotic cells.

BT-20 cells displayed a decrease in cell viability in addition to increased apoptotic cells when exposed to 0.223 µM, 0.167 µM and 0.112 µM ESE-16 for 48 hours, compared to DMSO (S1 Table). A significant increase in apoptotic cells were observed in MCF-7 cells exposed to 0.313 µM (54.24±2.52%) and 0.235 µM (25.25±2.00%) ESE-16, compared to DMSO (4.66±3.18%). MDA-MB-231 cell viability were significantly decreased with a concurrent increase in apoptosis following a 48-hour exposure to 0.274 µM, 0.206 µM and 0.137 µM, compared to the vehicle control (Figure S1).

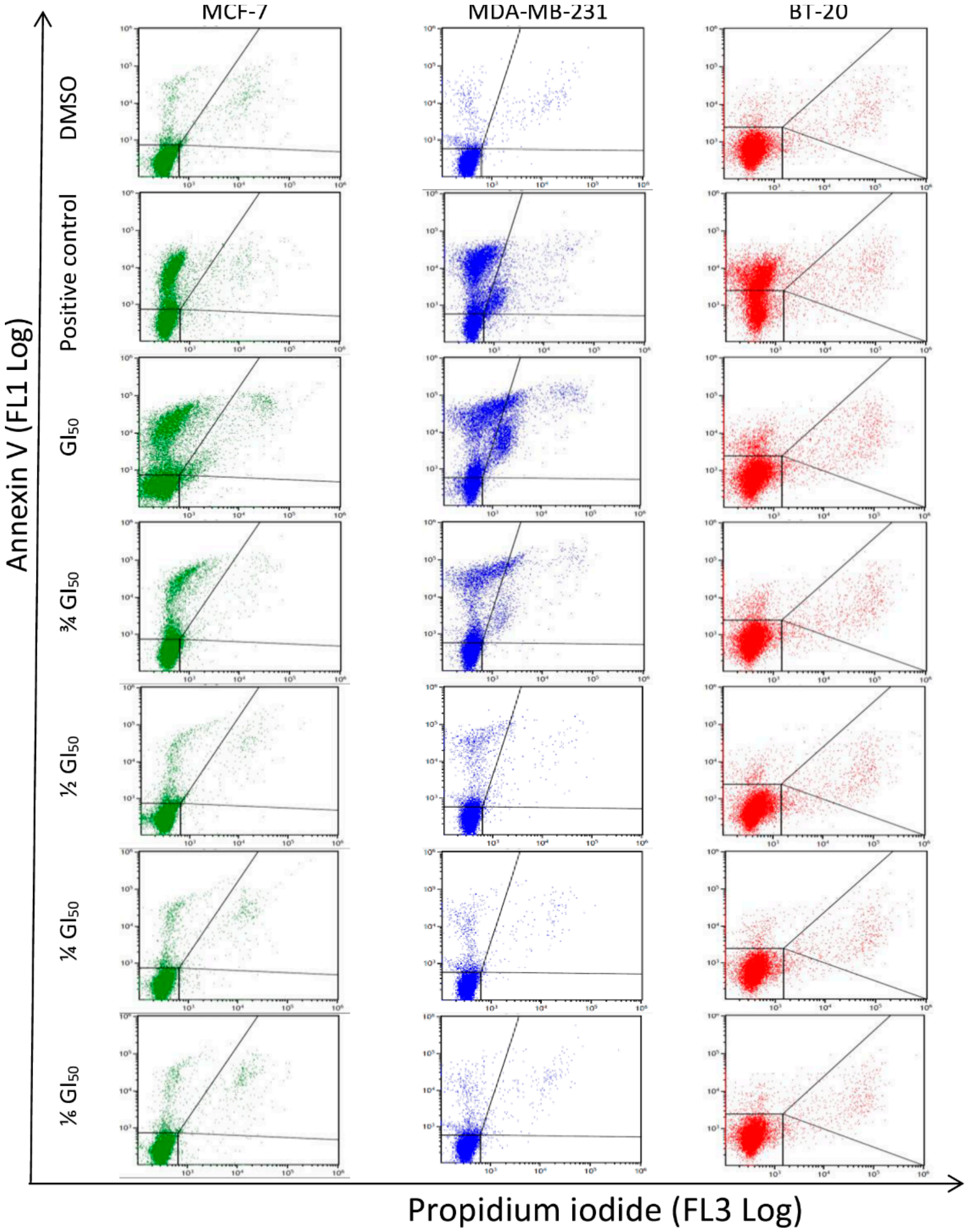

Figure S1: Apoptotic cell death in MCF-7-, MDA-MB-231- and BT-20 cells exposed to an ESE-16 dose curve as illustrated by flow cytometric dot plots of annexin V-FITC. Apoptotic cells were labelled with annexin V-FITC (FL1 Log) and were plotted against necrotic cells stained with propidium iodide (FL3 Log). Cells were propagated in growth media only (negative control), exposed to a DMSO as a vehicle control or exposed to a positive apoptotic control. Additionally, cells were exposed to an ESE-16 compound dose curve.

The lowest ESE-16 dose that significantly increased apoptotic cell death was determined at 0.167 µM, 0.235 µM and 0.137 µM in BT-20-, MCF-7- and MDA-MB-231 cells, respectively (Figure S2). These results were taken into consideration (with results obtained from cell cycle analysis) when the doses that were used to sensitize the cells prior to radiation were selected.

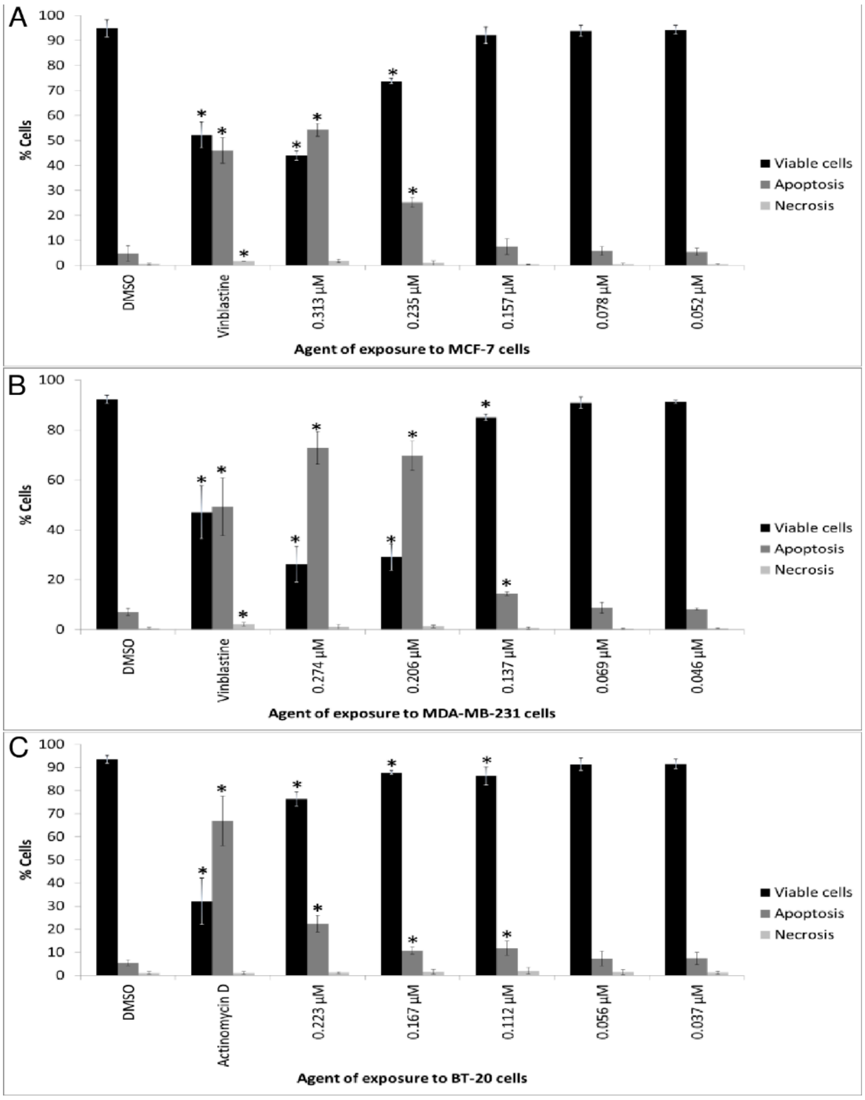

Figure S2: Graphical representation of annexin V-FITC quantification in MCF-7-, MDA-MB-231- and BT-20 cells exposed to an ESE-16 dose curve. MCF-7- (A), MDA-MB-231- (B) and BT-20 cells (C) were exposed to an ESE-16 dose curve. The lowest concentrations that significantly increased apoptosis were measured to be 0.235 µM, 0.137 µM and 0.167 µM ESE-16 for MCF-7-, MDA-MB-231- and BT-20 cells, respectively. Three biological repeats were completed for dose curves. The averages of viable, apoptotic and necrotic cells are displayed on the bar graph and the standard deviation is indicated by T-bars. A *P*-value <0.05 was considered statistically significant and are indicated with an *.

Table S1: Annexin V-FITC quantification in MCF-7-, MDA-MB-231- and BT-20 cells exposed to compound dose-response curves. Data were acquired and analyzed using Kaluza Analysis software version 1.5 (FL, USA) and an ANOVA-single factor model. Statistical significance was calculated using a two-tailed Student's *t*-test. The averaged percentage of three biological repeats ± standard deviations are displayed for viable- and apoptotic cells.

	Viable cells			Apoptosis		
	**MCF-7**	**MDA-MB-231**	**BT-20**	**MCF-7**	**MDA-MB-231**	**BT-20**
Medium only	93.99±2.56	92.53±3.40	91.69±2.90	5.50±2.34	6.61±3.22	7.03±2.96
DMSO	94.84±3.50	92.35±1.68	93.45±1.77	4.66±3.18	7.05±1.50	5.46±1.25
Positive control	52.20±5.13	47.03±10.59	32.01±10.04	46.06±5.06	49.38±11.54	66.89±10.60
GI_{50}	43.94±1.90	26.11±7.22	76.33±3.16	54.24±2.52	72.84±6.52	22.49±3.58
¾ GI_{50}	73.73±1.03	28.97±5.32	87.74±0.72	25.25±2.00	69.75±5.88	10.74±1.59
½ GI_{50}	92.10±3.23	85.05±1.25	86.22±3.89	7.55±3.13	14.35±0.85	11.76±3.12
¼ GI_{50}	93.83±2.15	90.87±2.23	91.35±2.71	5.75±1.67	8.77±2.07	7.26±3.11
⅙ GI_{50}	94.24±1.80	91.32±0.69	91.48±2.13	5.42±1.48	8.18±0.49	7.35±2.64

S3. Compound dose-response curve: Cell cycle analysis via flow cytometry.

The ability of breast cancer cells to progress through the cell cycle following exposure to an ESE-16 dose curve (GI_{50}, ¾ GI_{50}, ½ GI_{50}, ¼ GI_{50} or ⅙ GI_{50}) were examined to determine the lowest dose of the compounds that affect cell proliferation. These doses were used to sensitize the cells prior to radiation in order to determine whether an additive effect can be obtained in the combination therapy. Cell cycle analysis was performed 48 hours after exposure via flow cytometry employing propidium iodide.

Breast cancer cells were exposed to DMSO as a vehicle control and showed no significant difference in cell cycle distribution compared to cells propagated in growth medium only. As positive method controls BT-20 cells were exposed to actinomycin D, while MCF-7- and MDA-MB-231 cells were exposed to vinblastine. The positive apoptotic controls displayed a significant increase in sub-G_1 apoptotic cells with a decrease in the G_1 population (Figure S3 and S4, Table S2).

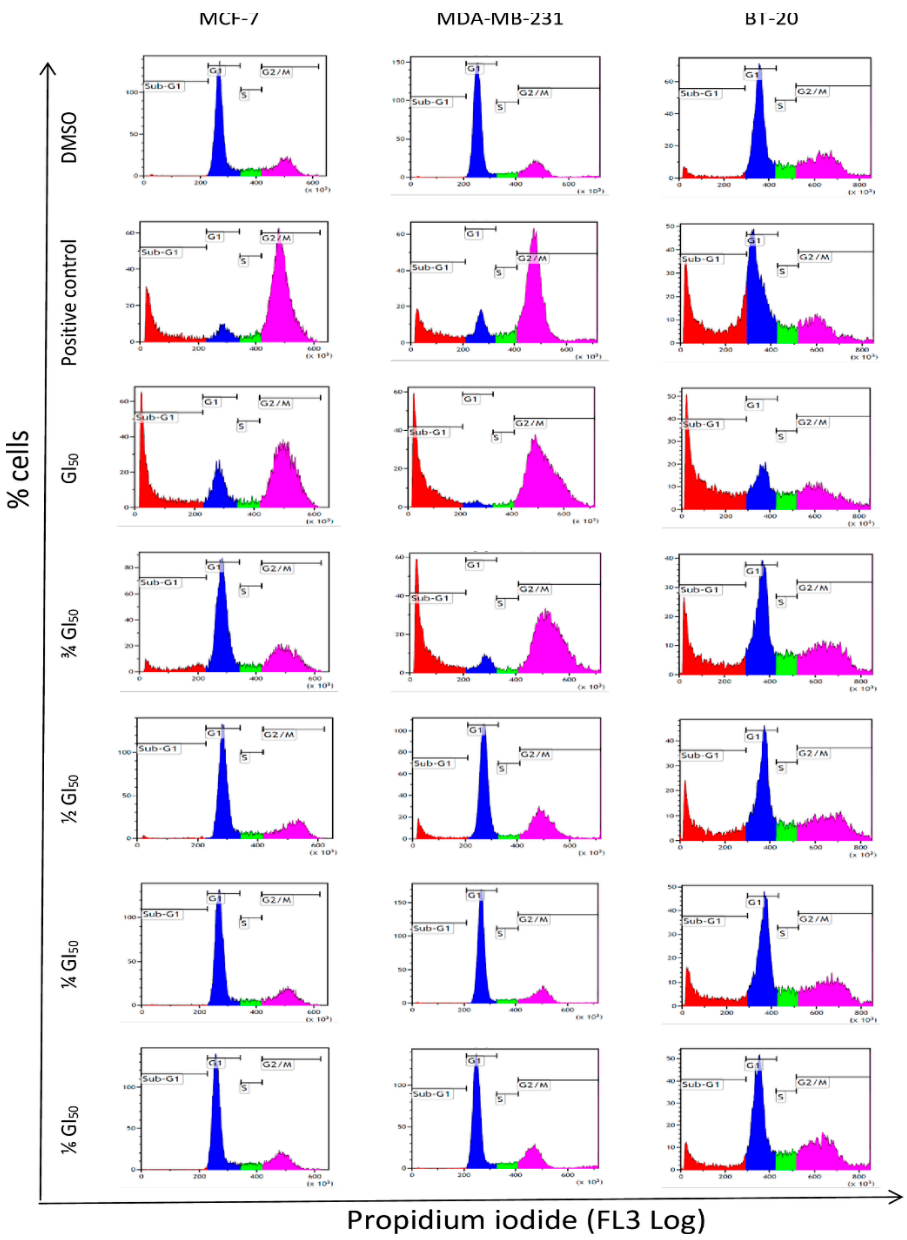

Figure S3: Histograms representing the cell cycle distributions of MCF-7-, MDA-MB-231- and BT-20 cells exposed to an ESE-16 compound dose curve. Histograms were obtained by plotting the number of cells against propidium iodide (FL3 Lin). Normal cell cycle distributions were presented in cells propagated in medium only (negative control) and cells exposed to DMSO (vehicle control). The positive apoptotic controls significantly increased the sub-G_1 phase. Cells were exposed to an ESE-16 dose curves to determine the lowest dose of each compound that significantly increase apoptotic cell death.

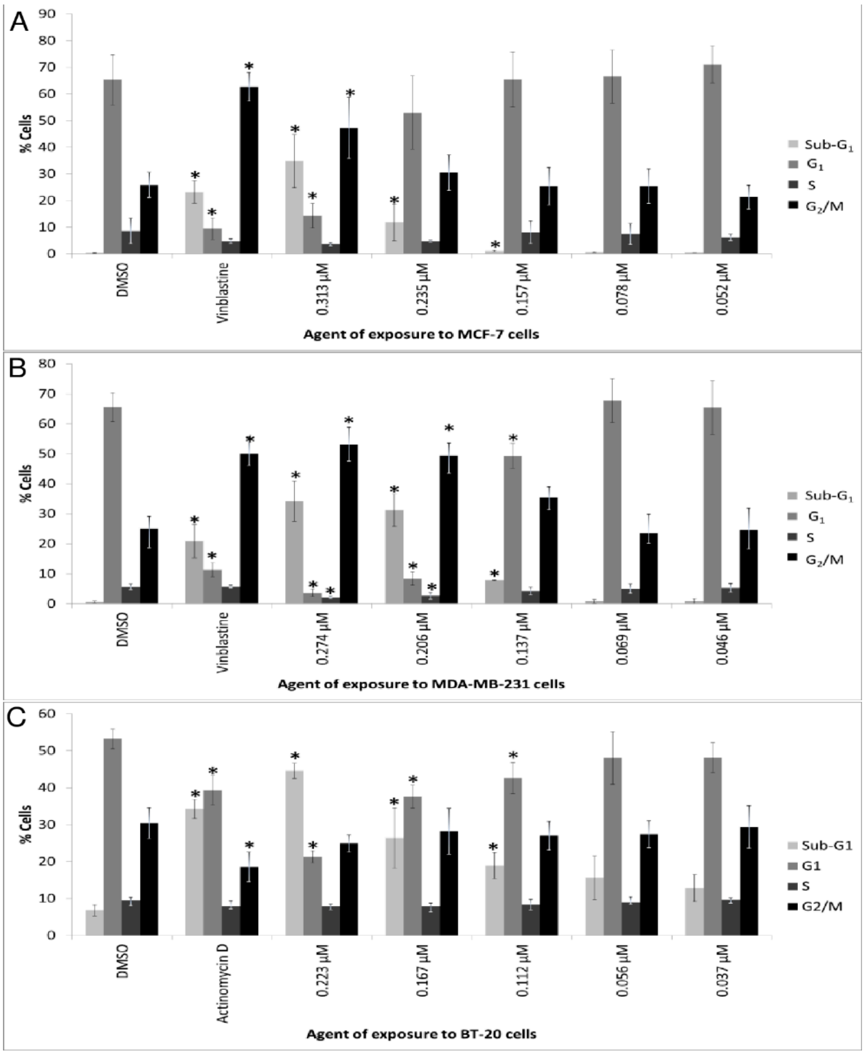

Figure S4: Graphical representation of the cell cycle analysis of MCF-7-, MDA-MB-231- and BT-20 cells exposed to ESE-16 dose curves. The lowest dose that significantly increased the sub-G_1 phase in MCF-7- (A), MDA-MB-231- (B) and BT-20 cells (C) were calculated to be 0.157 µM, 0.137 µM and 0.112 µM, respectively. Bars indicate the mean of three biological repeats, with standard deviations represented by T-bars. Statistically significant differences are indicated with * (P <0.05).

Table S2: Flow cytometric analysis of MCF-7-, MDA-MB-231- and BT-20 cells exposed to compound dose-response curves. Kaluza Analysis software version 1.5 (FL, USA) was used to acquire the data followed by analysis using an ANOVA-single factor model. A two-tailed Student's *t*-test was used to calculate statistical significance. The averaged percentage of three biological repeats ± standard deviations) are displayed for each phase of the cell cycle.

	**Sub-G$_1$**			**G$_1$**			**S**			**G$_2$/M**		
	MCF-7	MDA-MB-231	BT-20	MCF-7	MDA-MB-231	BT-20	MCF-7	MDA-MB-231	BT-20	MCF-7	MDA-MB-231	BT-20
Mediu	0.44±0.12	0.48±0.42	9.85±3.54	66.25±9.57	66.82±8.96	50.90±5.48	8.86±4.67	5.63±2.33	9.86±0.67	24.44±5.12	23.86±6.49	29.36±4.81
DMSO	0.33±0.10	0.62±0.32	6.77±1.47	65.28±9.39	65.57±4.75	53.23±2.63	8.59±4.69	5.62±0.93	9.55±1.51	25.79±4.73	25.11±3.99	30.41±4.13
Positive	23.17±4.18	20.82±5.52	34.22±2.61	9.43±4.08	11.38±2.35	39.30±3.98	4.72±0.92	5.74±0.50	7.86±0.63	62.65±5.36	50.10±5.54	18.57±4.03
GI$_{50}$	34.81±9.96	34.22±6.75	44.55±2.07	14.32±4.61	3.60±1.17	3.60±1.17	3.61±0.59	2.08±0.30	7.81±0.87	47.23±11.49	53.05±5.88	24.91±2.31
¾ GI$_{50}$	11.81±6.92	31.18±5.35	26.38±8.16	52.97±13.71	8.41±2.23	37.60±3.09	4.70±0.42	2.64±1.03	7.81±1.45	30.46±6.66	49.41±4.16	28.17±6.18
½ GI$_{50}$	1.05±0.32	7.83±0.08	18.90±3.54	65.47±10.22	49.33±4.15	42.56±4.19	8.07±4.23	4.21±1.27	8.32±1.46	25.35±7.02	35.47±3.44	27.00±3.93
¼ GI$_{50}$	0.62±0.18	0.83±0.67	15.60±5.86	66.54±10.05	67.81±7.31	48.03±7.11	7.47±3.93	5.01±1.50	8.89±0.42	25.33±6.41	23.56±6.33	27.39±3.67
⅙ GI$_{50}$	0.45±0.01	0.87±0.92	12.82±3.64	71.07±6.92	65.46±9.00	48.12±4.04	6.14±1.25	5.31±1.48	9.68±0.94	21.25±4.44	24.56±7.18	29.32±5.75

Cell cycle analysis and Annexin V studies revealed 0.137 μM ESE-16 to be the lowest dose to significantly increase apoptotic cell death in MDA-MB-231 cells, thus this dose was used to sensitize MDA-MB-231 cells to radiation. Cell cycle analysis of ESE-16 exposed MCF-7 cells displayed a significant increase in the sub-G1 phase following exposure to 0.157 μM, however apoptosis was only significant at 0.235 μM as quantified by annexin V. Thus 0.157 μM was used to presensitize MCF-7 cells 24 h prior to radiation. The lowest dose that significantly increased annexin V staining in ESE-16 exposed BT-20 cells was 0.167 μM, but the sub-G1 phase was significantly increased at 0.112 μM. Thus 0.112 μM ESE-16 was used to presensitize BT-20 cells.

S4. Radiation dose-response curve: Flow cytometric quantification of apoptosis induction.

The design behind the study necessitated the determination of a low radiation dose that significantly induced apoptosis. In order to do so, MCF-7-, MDA-MB-231- and BT-20 cells were exposed to a radiation dose curve (0 Gy, 2 Gy, 4 Gy, 6 Gy, 8 Gy and 10 Gy, respectively). The selected radiation dose was used in all subsequent radiosensitization experiments (Figure S5 and S6, Table S3).

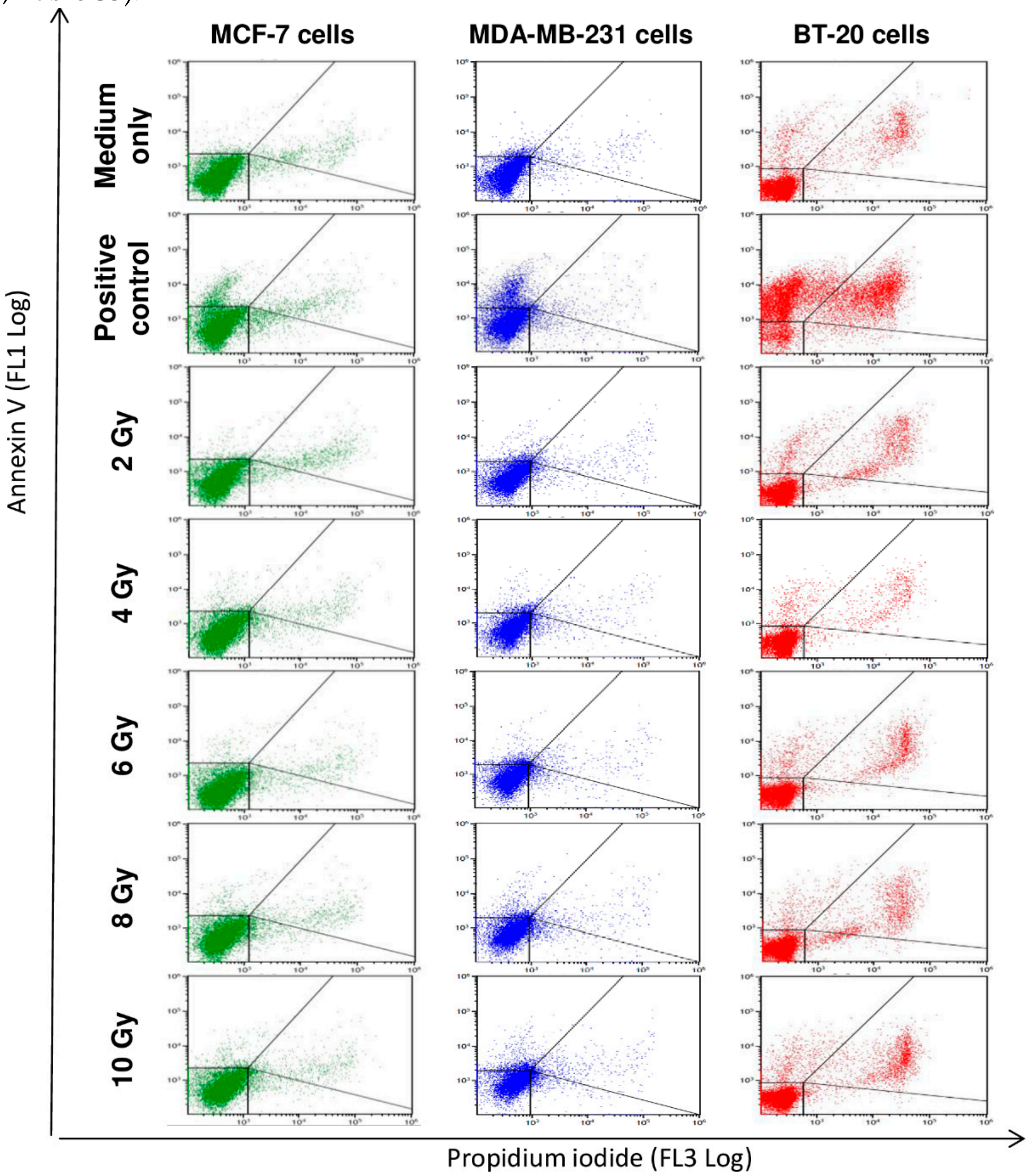

Figure S5: Annexin V-FITC flow cytometric dot plots of breast cancer cells exposed to a radiation dose range. Apoptotic cells were stained with annexin V-FITC (FL1 Log) and were plotted against propidium iodide (FL3 Log) that measured cells in late apoptosis and necrosis. MCF-7-, MDA-MB-231- and BT-20 cells were propagated in medium only (negative control), exposed to vinblastine (MCF-7- and MDA-MB-231 cells) or actinomycin D (BT-20 cells) as positive controls for apoptosis and a radiation dose curve: 2 gray (Gy), 4 Gy, 6 Gy , 8 Gy, 10 Gy.

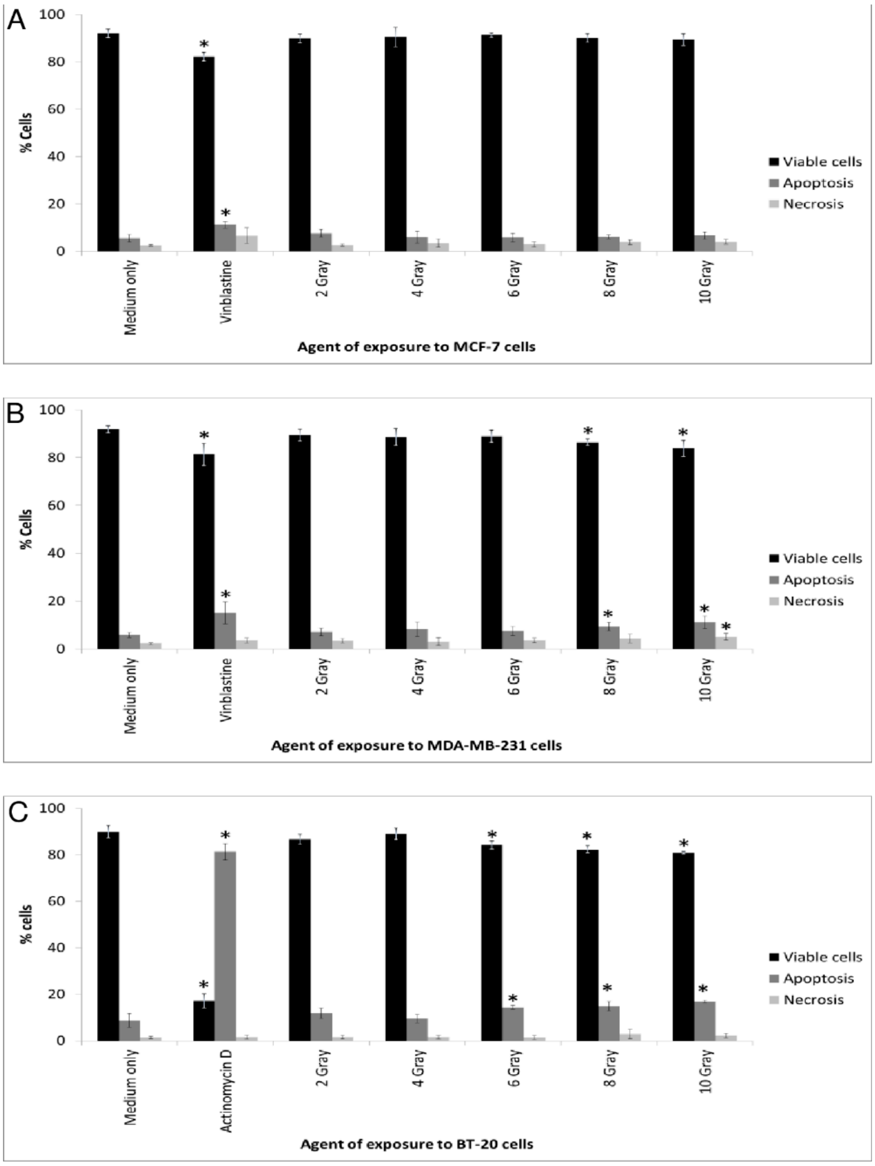

Figure S6: Graphical representation of the annexin V-FITC analysis of MCF-7-, MDA-MB-231- and BT-20 breast cancer cells exposed to a radiation dose range. MCF-7 cells displayed no significant increase in apoptosis induction upon radiation exposure (A). The lowest radiation dose that significantly induced apoptosis was calculate at 8 Gy for MDA-MB-231 cells (B) and 6 Gy for BT-20 cells (C). The averages of three biological repeats are displayed on the bar graph with standard deviation represented by T-bars. Statistically significant differences are indicated with * (*P*-value <0.05).

Table S3: Annexin V-FITC analysis of MCF-7-, MDA-MB-231- and BT-20 breast cancer cells following exposure to a radiation dose range. Kaluza Analysis software version 1.5 (FL, USA) was used to generate the statistical data. The ANOVA-single factor model was used to analyze the data and statistical significance was calculated using a two-tailed Student's *t*-test. The averaged percentage ± standard deviations of three biological repeats are displayed for viable- and apoptotic cells.

	**Viable cells**			**Apoptosis**		
	**MCF-7**	**MDA-MB-231**	**BT-20**	**MCF-7**	**MDA-MB-231**	**BT-20**
Medium only	92.03±1.88	91.88±1.48	89.97±2.62	5.50±1.62	5.85±1.08	8.67±3.01
Positive control	82.23±1.75	81.42±4.6	17.16±3.10	11.18±1.46	15.08±4.63	81.37±3.48
2 Gy	89.87±1.85	89.49±2.46	86.67±2.21	7.59±1.56	7.11±1.56	11.81±2.20
4 Gy	90.49±4.14	88.68±3.51	88.99±2.50	6.02±2.50	8.31±2.91	9.51±1.75
6 Gy	91.3±0.91	88.99±2.47	84.20±1.64	5.77±1.78	7.47±1.79	14.42±0.82
8 Gy	90.11±1.76	86.42±1.34	82.34±1.51	6.10±0.77	9.24±1.70	14.84±2.05
10 Gy	89.47±2.45	83.85±3.43	80.91±0.47	6.65±1.50	11.10±2.65	16.92±0.55

Results obtained from the quantification of annexin V-FITC staining revealed the lowest radiation doses to increase apoptosis to be 6 Gy in BT-20 cells and 8 Gy in MDA-MB-231 cells. MCF-7 cells revealed no significant increase in annexin V-FITC staining following radiation exposure. These results were used in conjunction with results obtained from cell cycle analysis in order to determine the radiation dose that were used in all subsequent experiments.

S5. Radiation dose response curve: Flow cytometric analysis of cell cycle progression.

MCF-7-, MDA-MB-231- and BT-20 cells were stained with propidium iodide following exposure to a radiation dose curve (0 Gy, 2 Gy, 4 Gy, 6 Gy, 8 Gy and 10 Gy, respectively). The results obtained from this experiment indicated the effect different radiation doses have on the cell cycle distribution of three breast cancer cell lines. Additionally, the induction of cell death (cells present in the sub-G_1 phase) was calculated and used to select a radiation dose that was used for all subsequent experiments (Figure S8, Table S4).

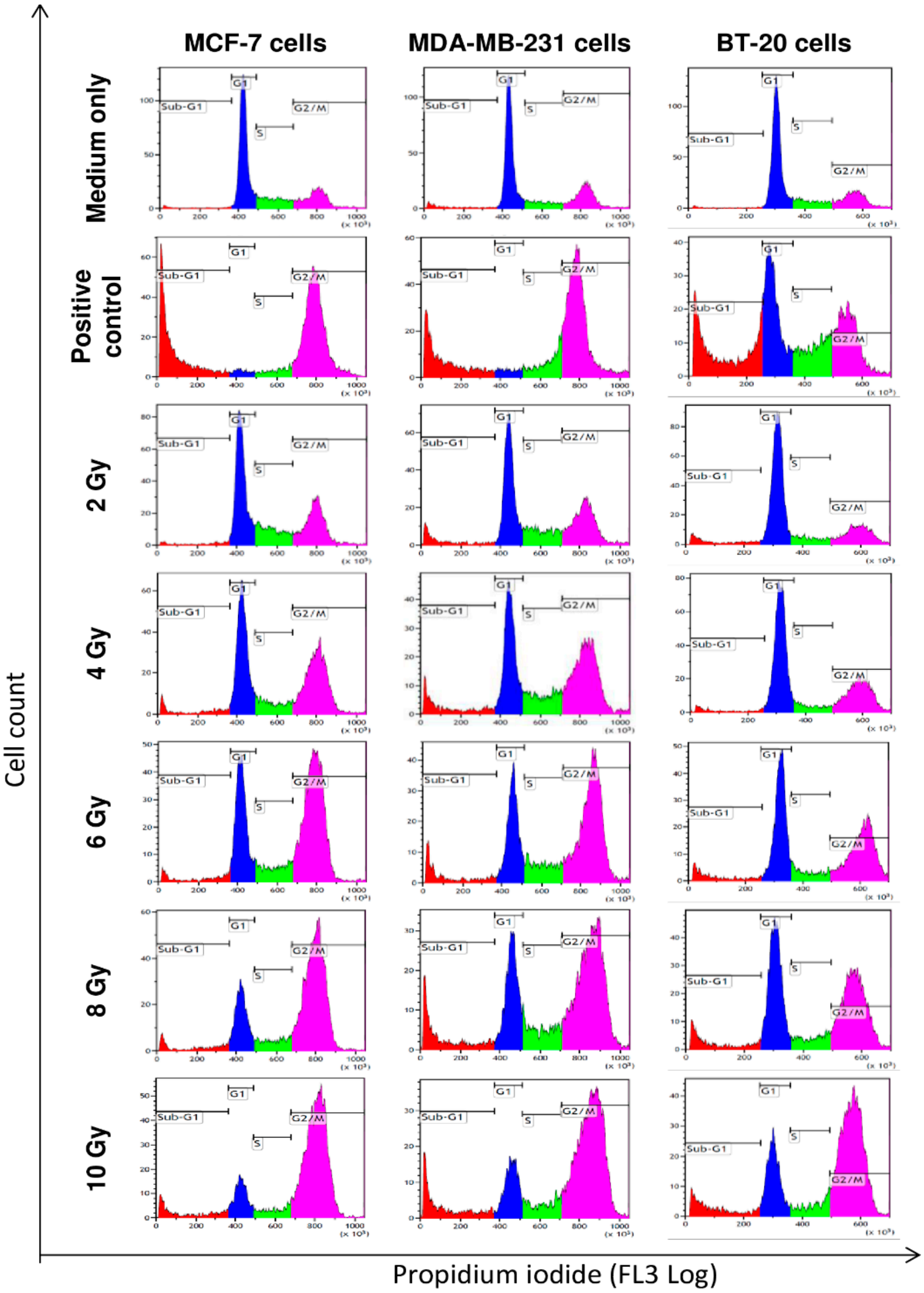

Figure S7: Histograms representing the cell cycle distributions of MCF-7-, MDA-MB-231- and BT-20 cells exposed to a radiation dose curve. The number of cells was plotted against propidium iodide (FL3 Lin). Normal cell cycle distributions were presented in cells propagated in medium only (negative control). Increased cell numbers in the sub-G_1 phase were observed in the positive apoptotic controls (vinblastine for MCF-7- and MDA-MB-231 cells, and actinomycin D for BT-20 cells). Alterations in the cell cycle distributions were observed in cells exposed to the different radiation doses.

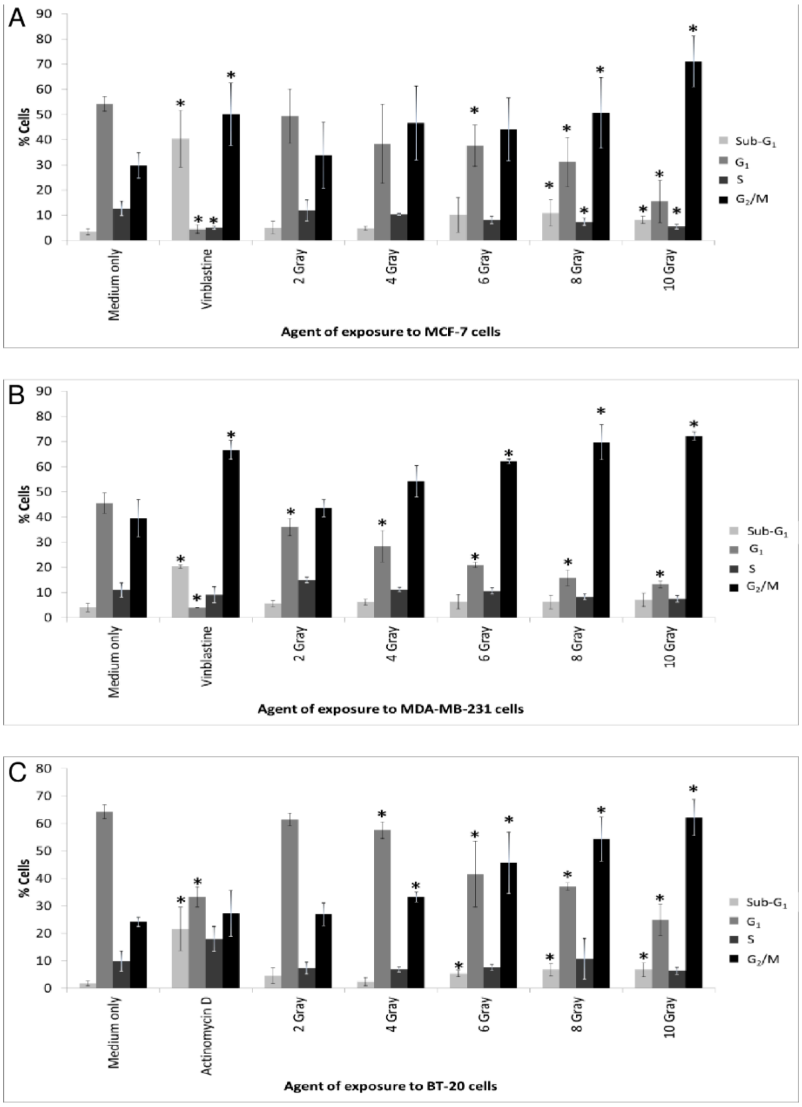

Figure S8: Graphical representation of the cell cycle analysis of MCF-7-, MDA-MB-231- and BT-20 cells exposed to a radiation dose range. MDA-MB-231 cells displayed no significant increase in the sub-G_1 phase of the cell cycle upon exposure to the radiation dose curve. The lowest radiation dose that significantly increased the sub-G_1 phase in MCF-7 cells was 8 Gy and 6 Gy in the BT-20 cell line. Bars indicate the mean of three biological repeats, with standard deviations represented by T-bars. Statistically significant differences are indicated with * (P-value <0.05).

Table S4: Cell cycle analysis of three breast cancer cell lines after exposure to a radiation dose-response curve. Data were generated using Kaluza Analysis software version 1.5 (FL, USA). Data were analyzed using ANOVA-single factor model and a two-tailed Student's *t*-test. The averaged percentage ± standard deviations of three biological repeats are displayed for cells present in the different phases of the cell cycle.

	Sub-G_1			G_1			S			G_2/M		
	MCF-7	MDA-MB-231	BT-20	MCF-7	MDA-MB-231	BT-20	MCF-7	MDA-MB-231	BT-20	MCF-7	MDA-MB-231	BT-20
Medium only	3.46±1.19	4.05±1.67	1.72±0.91	54.26±2.89	45.48±4.06	64.26±2.44	12.69±2.84	10.95±2.78	9.87±3.69	29.73±5.04	39.53±7.34	24.23±1.63
Positive control	40.36±11.31	20.31±0.67	21.57±7.87	4.51±1.70	3.99±0.08	33.21±3.69	5.08±0.57	9.09±3.16	17.99±4.52	50.13±12.44	66.66±3.75	27.29±8.40
2 Gy	5.03±2.57	5.59±1.23	4.47±2.86	49.35±10.73	35.99±3.38	61.44±2.24	11.92±4.19	14.94±1.08	7.32±2.11	33.81±13.11	43.52±3.48	26.89±4.16
4 Gy	4.79±0.77	6.19±1.12	2.36±1.50	38.33±15.71	28.42±6.27	57.50±3.01	10.35±0.38	11.18±0.80	6.85±0.86	46.66±14.72	54.22±6.30	33.26±1.79
6 Gy	10.13±6.92	6.32±2.85	5.32±1.17	37.70±8.25	20.96±1.01	41.50±11.97	8.14±1.46	10.57±1.26	7.52±1.13	44.11±12.52	62.14±0.88	45.71±11.12
8 Gy	10.93±5.18	6.25±2.68	6.67±2.26	31.11±9.86	15.80±3.19	37.01±1.35	7.35±1.40	8.27±1.17	10.71±7.46	50.74±13.95	69.74±6.99	54.34±8.09
10 Gy	8.19±1.43	7.09±2.65	6.70±2.52	15.43±8.50	13.25±1.41	24.83±5.75	5.45±0.88	7.49±1.31	6.35±1.29	71.03±10.08	72.17±1.67	62.11±6.53

The cell cycle analysis revealed that the lowest radiation dose that increased the sub-G_1 phase was 6 Gy for the BT-20 cell line and 8 Gy for the MCF-7 cell line. No statistically significant increase in sub-G_1 was observed in the MDA-MB-231 cells upon exposure to the radiation dose curve. However, annexin V-FITC results did indicate that apoptosis was significantly increased in the MDA-MB-231 cells after 8 Gy radiation. This may suggest that apoptosis is induced in MDA-MB-231 cells present in the G_2/M phase (metaphase block) after 8 Gy radiation. Thus, in order to keep the radiation dose delivery protocol constant 8 Gy was selected to be used in all the cell lines for the subsequent radiosentisitzation experiments.

Table S5: Cell cycle analysis of BT-20-, MCF-7- and MDA-MB-231 cells exposed to the various treatment conditions. Data were generated and analyzed, using Kaluza Analysis software version 1.5 (FL, USA) and the ANOVA-single factor model, respectively. A two-tailed Student's *t*-test was used to calculate statistical significance. The averaged percentage and the standard deviations of three biological repeats are summarized in the table below.

		DMSO	Positive control	ESE-16	8 Gy radiation	ESE-16 + radiation
		Averaged percentage ± standard deviation				
Sub-G_1	BT-20	7.87±3.74	32.84±3.59	23.77±0.75	20.49±5.37	25.75±1.01
	MCF-7	1.52±0.07	33.04±11.76	2.12±0.04	3.89±0.24	6.27±0.72
	MDA-MB-231	1.31±0.45	37.65±9.50	2.41±0.01	5.71±2.86	5.90±2.22
G_1	BT-20	57.17±3.49	39.31±5.54	48.29±0.64	33.13±11.34	28.99±0.67
	MCF-7	51.01±3.79	5.19±1.18	47.74±2.32	25.81±2.60	28.05±6.58
	MDA-MB-231	54.22±0.91	5.39±1.05	48.47±2.05	32.35±4.69	30.90±1.96
S	BT-20	7.33±1.48	8.95±3.13	7.88±1.36	7.01±1.95	9.15±0.35
	MCF-7	7.88±1.29	3.97±0.51	8.99±1.42	5.00±0.81	5.02±0.41
	MDA-MB-231	10.30±3.02	7.58±3.66	10.40±2.84	7.01±1.85	7.81±1.95
G_2/M	BT-20	27.87±4.74	20.04±5.87	20.41±2.14	47.76±10.31	36.64±0.43
	MCF-7	39.56±4.14	63.87±11.49	40.47±3.52	62.98±6.61	61.38±6.94
	MDA-MB-231	34.16±3.33	49.34±10.91	38.69±4.89	54.89±9.06	55.35±5.77

Table S6: Apoptotic induction measured by annexin V-FITC quantification in BT-20-, MCF-7- and MDA-MB-231 cells exposed to various treatment conditions. Data were generated and analyzed using Kaluza Analysis software version 1.5 (FL, USA) and the ANOVA-single factor model, respectively. A two-tailed Student's *t*-test was used to calculate statistical significance. The averaged percentage ± standard deviations for viable, apoptotic and necrotic cells are summarized in the table below.

		DMSO	Positive control	ESE-16	8 Gy radiation	ESE-16 + radiation
		Averaged percentage ± standard deviation				
Viable cells	BT-20	90.68±0.24	16.90±2.24	86.41±1.49	85.73±1.86	83.35±0.76
	MCF-7	93.93±1.31	54.74±8.89	90.79±4.99	84.60±2.75	73.68±2.22
	MDA-MB-231	94.98±0.67	47.80±10.01	89.93±1.81	84.81±1.41	80.58±2.10
Apoptosis	BT-20	8.76±0.33	82.38±2.29	12.56±1.01	13.60±1.53	15.83±1.25
	MCF-7	4.98±1.39	43.30±7.85	8.79±4.94	14.77±2.99	25.39±1.84
	MDA-MB-231	4.70±0.56	50.37±10.77	9.69±1.89	14.37±1.70	18.76±2.04
Necrosis	BT-20	0.56±0.32	0.72±0.19	1.03±0.82	0.67±0.34	0.82±0.49
	MCF-7	1.09±0.44	1.96±1.08	0.41±0.27	0.63±0.39	0.93±0.52
	MDA-MB-231	0.31±0.12	1.83±0.77	0.37±0.08	0.83±0.32	0.66±0.22

S6. Long term cellular proliferation in treated MCF-7-, MDA-MB-231- and BT-20 cells.

Table S7: Cellular proliferation measure 14 days after radiation in MCF-7-, MDA-MB-231- and BT-20 cells following exposure to various treatment conditions. The crystal violet stain was solubilized and the optical density was measured at 570 nm. Cellular proliferation is expressed as fold change and was calculated relative to the vehicle control taken as 1. In the table below the averaged fold change (FC) and standard deviations (SD) of three biological repeats (n=3) are displayed. A two-tailed Student's *t*-test was used to calculate statistically significant differences (*P*-value <0.05) using the vehicle control and individual treatments conditions as baselines.

		DMSO	Vinblastine	Compound	4 Gy radiation	Combination
MCF-7	FC± SD	1	0.08±0.01	0.77±0.05	0.19±0.03	0.14±0.01
	P-value	-	**6.24E-10**	**1.12E-03**	**7.55E-07**	**7.93E-09**
MDA-MB-231	FC ± SD	1	0.09±3.58E-03	0.89±0.06	0.33±0.02	0.18±0.02
	P-value	-	**1.56E-10**	**0.04**	**8.52E-07**	**2.79E-07**
BT-20	FC ± SD	1	0.04±0.01	1.00±0.09	0.23±0.01	0.17±0.01
	P-value	-	**5.29E-10**	0.99	**9.13E-08**	**7.04E-08**
		**Compared to compound**				
MCF-7	FC ± SD			1		0.19±0.01
	P-value			-		**5.63E-09**
MDA-MB-231	FC ± SD			1		0.20±0.03
	P-value			**-**		**2.00E-06**
BT-20	FC ± SD			1		0.18±0.03
	P-value			-		**6.94E-07**
		**Compared to 4 Gy radiation**				
MCF-7	FC ±S D				1	0.78±0.10
	P-value				**-**	**1.93E-02**
MDA-MB-231	FC ± SD				1	0.55±0.10
	P-value				**-**	**1.25E-03**
BT-20	FC ± SD				1	0.75±0.05
	P-value				-	**9.52E-04**

S7. Micronuclei quantification

Table S8: Micronuclei quantification in breast cancer cells pre-treated with ESE-16. MCF-7- and MDA-MB-231- and BT-20 cells were exposed to ESE-16, 8 Gy radiation and ESE-16/radiation. Micronuclei quantification followed 2- and 24 hours after radiation.

		**Number of micronuclei**	**MCF-7**		**MDA-MB-231**		**BT-20**	
			**2h**	**24h**	**2h**	**24h**	**2h**	**24h**
Cells presented with micronuclei	DMSO	0	481	484	471	477	452	456
		1	16	15	29	22	43	40
		2	3	1	1	2	6	3
		3	0	0	0	0	0	0
		4	0	0	0	0	0	0
		5	0	0	0	0	0	0
		>5	0	0	0	0	0	0
	ESE-16	0	478	482	471	476	444	425
		1	19	14	28	23	53	66
		2	3	4	1	1	3	7
		3	0	0	0	0	0	1
		4	0	0	0	0	0	0
		5	0	0	0	0	0	0
		>5	0	0	0	0	0	0
	8 Gy radiation	0	219	220	299	303	273	280
		1	226	244	145	146	162	176
		2	45	35	34	45	49	39
		3	8	1	21	6	12	6
		4	2	0	1	0	3	1
		5	0	0	0	0	1	0
		>5	0	0	0	0	0	0
	ESE-[illegible]	0	286	249	298	293	254	259
		1	102	188	144	137	160	164

		2	86	50	34	54	62	54
		3	18	11	24	15	16	17
		4	7	2	0	0	7	5
		5	1	0	0	0	1	1
		>5	0	0	0	0	0	1
